# Activation of PAA at the Fe–N_*x*_ Sites by Boron Nitride Quantum Dots Enhanced Charge Transfer Generates High-Valent Metal-Oxo Species for Antibiotics Degradation

**DOI:** 10.1021/acs.est.4c08224

**Published:** 2024-11-28

**Authors:** Shuo Li, Yalun Yang, Junfeng Niu, Heshan Zheng, Wen Zhang, Yoong Kit Leong, Jo-Shu Chang, Bo Lai

**Affiliations:** † College of Food and Bioengineering, Qiqihar University, Qiqihar 161006, China; ‡ College of Environmental Science and Engineering, North China Electric Power University, Beijing 102206, China; § John A. Reif, Jr. Department of Civil and Environmental Engineering, New Jersey Institute of Technology, Newark, New Jersey 07102, United States; ∥ Department of Chemical and Materials Engineering, Tunghai University, Taichung 407, Taiwan; ⊥ Research Center for Smart Sustainable Circular Economy, Tunghai University, Taichung 407, Taiwan; # Department of Chemical Engineering and Materials Science, Yuan Ze University, Chung-Li 320 Taiwan; ∇ Department of Environmental Science and Engineering, School of Architecture and Environment, Sichuan University, Chengdu 610065, China

**Keywords:** peracetic acid, high-valent iron-oxo species, Fe−N_*x*_ sites, selective attack

## Abstract

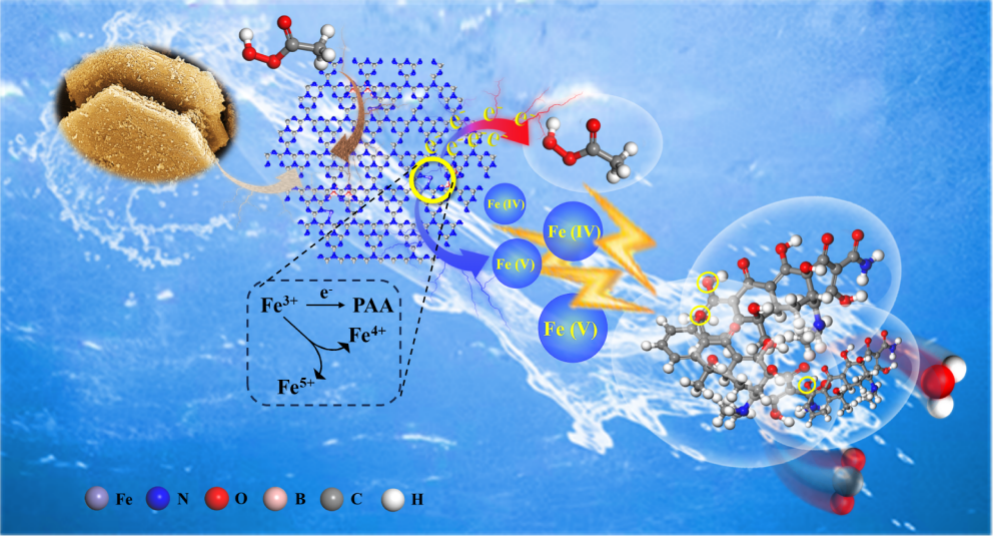

Advanced oxidation processes (AOPs) based on peracetic acid (PAA) offer a promising strategy to address antibiotic wastewater pollution. In this study, Fe-doped graphitic carbon nitride (g-C_3_N_4_) nanomaterials were used to construct Fe–N_*x*_ sites, and the electronic structure was tuned by boron nitride quantum dots (BNQDs), thereby optimizing PAA activation for the degradation of antibiotics. The BNQDs-modified Fe-doped g-C_3_N_4_ catalyst (BNQDs-FCN) achieved an excellent reaction rate constant of 0.0843 min^–1^, marking a 21.6-fold improvement over the carbon nitride (CN)-based PAA system. DFT calculations further corroborate the superior adsorption capacity of the Fe–N_*x*_ sites for PAA, facilitating its activation. Charge transfer mechanisms, with PAA serving as an electron acceptor, were identified as the source of high-valent iron-oxo species. Moreover, the BNQDs-FCN system preferentially targets oxygen-containing functional groups in antibiotic structures, elucidating the selective attack patterns of these highly electrophilic species. This research not only elucidates the pivotal role of high-valent iron-oxo species in pollutant degradation within the PAA-AOPs framework but also pioneers a wastewater treatment system characterized by excellent degradation efficiency coupled with low ecological risk, thereby laying the groundwork for applications in wastewater management and beyond.

## Abstract 2

Few studies focus on PAA activation to produce high-valent metal species. However, the BNQDs-FCN/PAA system here generates high-valent metal-oxo species that selectively attack oxygen-containing groups in antibiotics.

## Introduction

With the recent rapid growth of the livestock and medical industries, antibiotics are often found in water, including both surface and underground sources. These antibiotics can have negative impacts on aquatic ecosystems and organisms.^1^ They can interact with bacteria in the environment, allowing bacteria that would otherwise be sensitive to antibiotics to acquire antibiotic resistance. The horizontal transmission of antibiotic resistance genes makes clinical antibiotic treatment more difficult.^2^ In addition, antibiotics can disrupt the balance of microbiomes in bodies of water and cause other potentially toxic effects on human health and ecosystems, which could lead to a public health crisis.^3^ The entry of antibiotics into the environment causes a severe ecological risk because some of them are chemically stable and not easily biodegradable. Thus, there is an urgent need to develop more efficient and ecofriendly technologies for the comprehensive mitigation of organic pollutants to reduce their impact on aquatic ecosystems.

Compared to other advanced oxidation processes (AOPs), such as persulfate (PS)-AOPs and periodate (PI)-AOPs, peracetic acid (PAA)-AOPs is considered a promising method for degrading organic pollutants due to its low pH dependence, high bactericidal capacity, and minimal production of toxic byproducts.^4−7^ PAA-AOPs can produce highly reactive oxide species from a variety of activation modes such as UV treatment and interaction with transition metals. In the case of UV activation, UV energy can dissociate O–O groups of PAA to form hydroxy (•OH) and acetoxyl radicals (CH_3_C(O)O•), respectively.^8^ Transition metals are also commonly used to activate PAA-AOPs.^4^ The use of cobalt (Co(II)) activated the degradation of sulfamethoxazole (SMX) by PAA. SMX was efficiently removed even at low doses of Co (<1 μM) due to the powerful redox cycle of Co(III)/Co(II).^9^ Acetylperoxy radical (CH_3_C(O)OO•) was the main contributor to the degradation of SMX, while CH_3_C(O)O• may have had a lesser contribution. However, the light energy utilization of UV radiation is currently low, and the transmittance of wastewater has to be considered. There are also transition metals, such as Co, that face limitations such as resource scarcity, high cost, and toxicity.

Among transition metals, iron (Fe) is both more accessible and cost-effective due to its ubiquitous presence in rocks, soil, and water. Additionally, iron is less toxic and has a lower environmental impact.^10^ Studies have shown that iron effectively activates PAA. In the degradation of micropollutants using the Fe(II)-PAA system, the central carbon radicals (•CH_3_, CH_3_C(O)•, and CH_3_C(O)O•) and/or Fe(IV) have a critical function in the first stage of the degradation reaction, while •OH plays an important role in the second stage of the reaction.^11^ In contrast, for the degradation of pollutants with Fe(III)-PAA, experiments show that Fe(V) and Fe(IV) dominate the degradation process, with organic radicals contributing less significantly.^12^

High-valent iron-oxo species have been widely studied for their high reactivity and ability to decompose organic pollutants in a variety of pH environments.^13,14^ Additionally, heterogeneous activation can reduce secondary contamination and enable catalyst recycling, thereby reducing degradation costs compared to homogeneous activation.^15^ To construct a heterogeneous activation system, it is important to find a suitable carrier for Fe element. Recently, graphitic carbon nitride (g-C_3_N_4_), a nonmetallic layered material rich in nitrogen, has been widely used in advanced oxidation treatments for water due to its chemical stability, low cost, and ease of preparation.^16^ The nitrogen-containing moieties (N–, – NH_2_, and – NH−) in g-C_3_N_4_ behave as strong Lewis bases, making them well-suited for the deposition of nanoparticles and the inclusion of metal atoms.^15^ Consequently, the N-rich nature of g-C_3_N_4_ offers abundant N sites for forming robust metal–N complexes (M–N–C).^17^ The Fe–N_*x*_ active sites generated by the coordination of Fe and N maximize the use of metal atoms and improve their catalytic efficiency. Additionally, the special redox properties of the central iron atom have led to investigations into PMS activation using iron complexes connected to N-based ligands, resulting in the oxidation of organic matter by high-valent iron-oxo species via a nonradical pathway.^14^ It is possible that Fe coordination structures in N-rich environments also promote charge transfer of the central iron atom through PAA activation, producing high-valent iron-oxo species. Improving the charge transfer efficiency remains a key challenge for future research.

Modifying the catalyst with boron nitride quantum dots (BNQDs) is planned to enhance electron transfer in their catalysts. Boron nitride in its hexagonal form (h-BN) is a 2D semiconductor with a molecular structure similar to that of graphene. As such, h-BN has attracted much attention because it is both chemically and thermally stable, has good thermal conductivity and mechanical strength, and possesses distinctive electrical properties.^18^ BNQDs are of specific interest because when the size of semiconductor particles is reduced to the quantum dot level, they generate new features due to quantum confinement effects and prominent edge properties.^19^ BNQDs, spherical particles with a diameter of nanometers, are characterized by low toxicity, biocompatibility, a quantum confinement effect, a high surface-to-volume ratio, higher tunability than h-BN, and their facile formation of hybrid structures with other semiconductors.^18,20^ BNQDs-modified WO_3_ nano blocks can be used as photoelectrochemical hydrolysis catalysts, and it was found that the BNQDs enhanced the charge separation and hindered the charge complexation process.^21^ BNQDs have a strong electrostatic attraction to photogenerated holes because of the proximity of negatively charged oxygen-based moieties that enable the dissociation of photogenerated electron–hole pairs.^22,23^ Additionally, quantum confinement in the BNQDs gives them a higher surface–volume ratio, resulting in quantum dots with grain sizes smaller than the Bohr radius of the volume of excitation, which also produces an abundance of reactive sites for the target.^24,25^

This work introduced a novel method to activate PAA for the degradation of tetracycline (TC) antibiotics via the use of BNQDs to enhance the charge separation in a catalyst composed of g-C_3_N_4_ doped with Fe(III) (BNQDs-FCN). Several reaction parameters are evaluated to determine their effect on the ability of the BNQDs-FCN system to degrade TC. The mechanism behind high-valent iron-oxo species generation is analyzed with calculations based on density functional theory (DFT), and the selectivity of antibiotic degradation is elucidated. Also, the practicality and applicability of the system are evaluated. Also, a mechanism for the degradation of TC is determined, the degradation intermediates are simulated, and their toxicity is estimated. This work provides new insights into the use of PAA-AOPs for the effective degradation of organic pollutants.

## Materials and Methods

### Chemical Reagents

The details of the chemical reagents are provided in Text S1.

### Catalyst Preparation

BNQDs were prepared using the top-down liquid-phase ultrasound stripping method, and BNQDs-FCN were prepared using the pyrolysis method. Details are provided in Text S2.

### Experimental Details

The degradation process was carried out in a 250 mL beaker placed at room temperature (25 ± 2 °C) with a magnetic stirring speed of 400 rpm. Detailed experimental procedures are provided in Text S3.

### Methods

Details of the analytical and DFT calculation methods are provided in Texts S4 and S5.

## Results and Discussion

### Synthesis and Characterization of BNQDs-FCN

**Figure 1:**
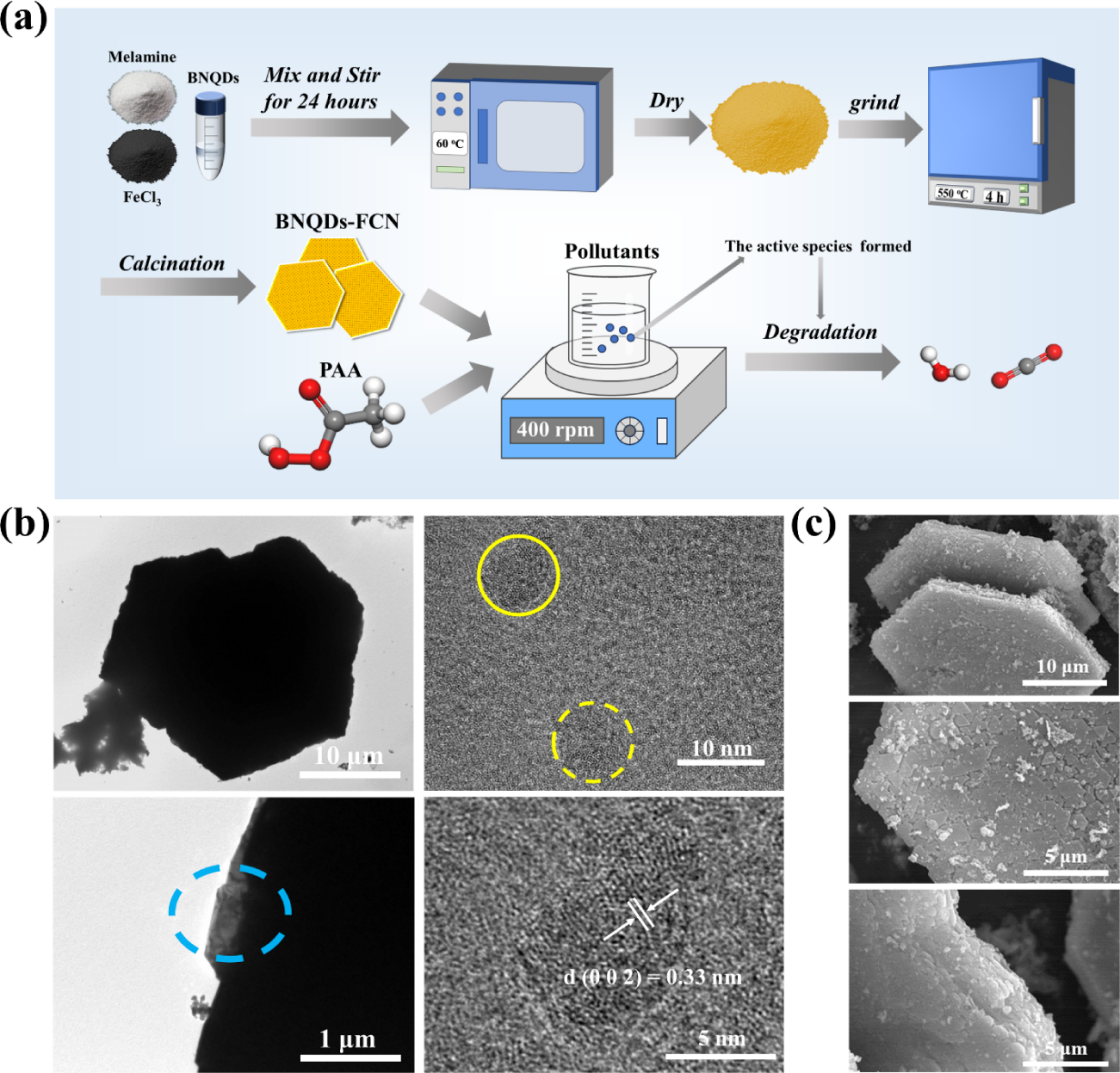
(a) Schematic representation of the preparation and catalytic degradation application of BNQDs-FCN, (b) HRTEM images, and (c) SEM images of BNQDs-FCN.

BNQDs-FCN were successfully prepared by pyrolysis and employed in catalytic pollutant degradation applications (Figure 1a). ICP-MS analysis revealed that the Fe content in BNQDs-FCN was approximately 27.34%. Figure S1a shows the TEM images of CN and FCN, in which the laminar structure of the catalyst becomes larger after doping with Fe. After modification with the BNQDs, this irregular laminar structure transforms into a well-defined hexagonal shape (Figure 1b). Further magnification of the blue circle region revealed the presence of nanodots (yellow circles in Figure 1b), and the *d*-spacing of their lattice stripes was determined to be 0.33 nm, matching the hexagonal pattern of the (002) crystal surface of BN.^26,27^ These results confirmed that the nanodots are BNQDs and also demonstrated successful doping of BNQDs. In addition, the lamellar structure of the catalyst was similarly observed by SEM (Figure 1c). Atomic force microscopy (AFM) (Figure S1b) showed the nanosheet morphology of the material. The synthesized BNQDs-FCN material possessed excellent fluorescence responsiveness and emitted bright blue light under an excitation wavelength of 365 nm, which indicates its superior photovoltaic performance and charge transfer (Figure S1c).

**Figure 2:**
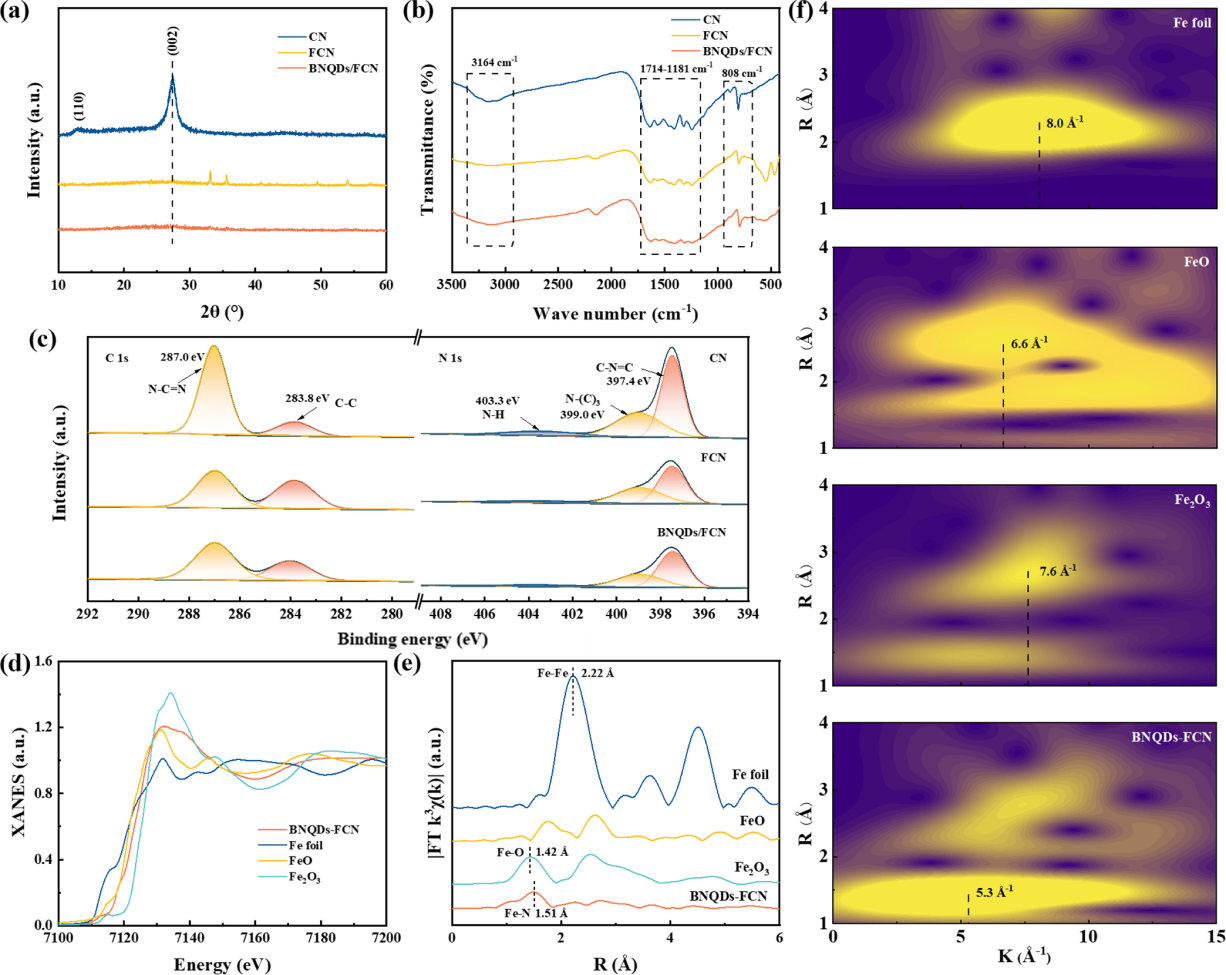
(a) XRD patterns, (b) FT-IR spectra, (c) XPS high-resolution spectrum of C 1s and N 1s, (d) XANES spectra at the Fe K-edge, (e) k^3^-weighted Fourier transform spectra, and (f) WT plot of Fe foil, FeO, Fe_2_O_3_, and BNQDs-FCN.

The two distinct peaks in the X-ray diffraction (XRD) pattern of CN at 13.1° and 27.4° represent the (002) and (100) crystal planes of g-C_3_N_4_, respectively (Figure 2a). The (002) and (100) planes represent the interlayer stacking and tri-s-thiazine units of the material, respectively.^28^ The XRD pattern of FCN reveals that the position of the (002) characteristic peak does not change significantly with Fe doping, but its intensity diminishes, which indicates the successful embedding of Fe into the g-C_3_N_4_ matrix.^29^ However, peaks at 33.1°, 35.6°, 40.8°, 49.4°, and 54.1°, which are typical diffraction peaks belonging to Fe_2_O_3_, were also observed, probably due to pyrolysis in an air atmosphere. Interestingly, the addition of BNQDs caused a nearly complete disappearance of these diffraction peaks of Fe_2_O_3_. Similarly, no peaks representing Fe crystals were observed, and these results suggest that Fe is primarily present in the composites in the form of Fe–N.^14^ In the Fourier-transform infrared (FT-IR) spectra of the materials, three absorption regions are readily apparent at 3164, 1714–1181, and 808 cm^–1^, which arise from N–H bond stretching, C=N and C–N bond stretching, and the bending vibration of the triazine structure, respectively (Figure 2b).^30−32^ As in the case of the XRD patterns, peaks belonging to Fe_2_O_3_ (548 and 463 cm^–1^) are apparent in the FT-IR spectrum of FCN, but these two peaks almost disappear in the BNQDs-FCN spectrum, where the presence of BNQDs promotes the formation of Fe–N. The gradual diminishing of the intensity of the peaks at 1714–1181 cm^–1^ for FCN and BNQDs-FCN relative to the pristine CN could be attributed to a greater presence of amorphous carbon caused by the coordination of Fe with the triazines in the FCN, which also indicates that Fe–N is present.^5^

In the high-resolution X-ray photoelectron spectrum (XPS) of C 1s, the characteristic peaks at 283.8 and 287.0 eV belong to C–C and hybridized carbon (N–C=N) bonds (Figure 2c).^33,34^ With the incorporation of Fe, the proportion of characteristic peaks belonging to N–C=N becomes smaller, which may be caused by the presence of Fe–N units affecting the molecular environment of nearby C atoms. Also shown is the XPS spectrum of N 1s, in which three characteristic peaks at 397.4, 399.0, and 403.3 eV can be observed, which represent the hybridized aromatic nitrogen of the triazine group (C–N=C), the tertiary nitrogen (N–(C)_3_), and amino (N–H) bonds, respectively.^5,30^ Similarly, the decrease in the proportion of characteristic peaks belonging to C–N=C proves that Fe–N groups are present. In the Fe spectrum in Figure S1d, Fe 2p peaks can be observed in the FCN and BNQDs-FCN catalysts, confirming the successful incorporation of Fe.^17^ In the spectrum B, the peak at 190.1 eV is attributed to the coordination of B–N (Figure S1e).^35^

X-ray absorption fine structure (XAFS) and extended XAFS (EXAFS) patterns were analyzed to study the atomic local structure and coordination of BNQTS-FCN. In the near-edge X-ray absorption structure (XANES) spectrum of the K-edge of Fe (Figure 2d), the absorption edge of BNQDs-FCN appeared between the absorption edge of Fe_2_O_3_ and FeO, indicating the presence of a Fe^δ+^ (2 < δ < 3) electronic structure.^36^ Consistent with the analysis of the XANES spectra, the XPS fine map of Fe shows that the introduction of BNQDs leads to an increase in the density of electrons near the Fe atoms, resulting in a modest decline in the valence state of Fe. In addition, the Fourier-transform EXAFS spectrum of BNQDs-FCN (Figure 2e) shows a peak at 1.51 Å that corresponds to the Fe–N bond. Unlike the coordination peaks of Fe–Fe in the Fe foil at 2.22 Å and of Fe–O in the Fe_2_O_3_ sample at 1.42 Å, no peaks belonging to Fe–Fe or Fe–O were apparent in the BNQDs-FCN spectrum, indicating that no iron oxides or clusters were present. This result further proves the dispersion of Fe atoms in the BNQDs-FCN catalyst. A wavelet transform contour map is presented in Figure 2f, in which Fe–N bonds are responsible for the peak intensity of BNQDs-FCN at 5.3 Å^–1^. As shown in Figure S1f-g, the coordination environment of Fe atoms in BNQDs-FCN was further analyzed by fitting the space with a crystallographic model from which the corresponding fitting parameters were obtained. The coordination number of Fe was found to be approximately 4 (Table S1), indicating that Fe and N atoms in BNQDs-FCN are arranged in a four-coordinate Fe–N_4_ configuration. These findings also suggest a well-dispersed distribution of Fe atoms within the catalyst.

### Performance Evaluation and Active Species Identification

**Figure 3:**
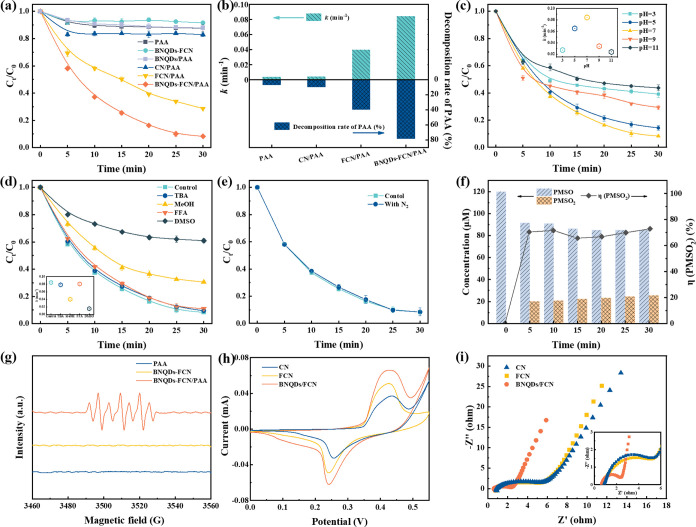
(a) Degradation of TC in different processes, (b) reaction rate constant and decomposition rate of PAA, (c) effect of pH on TC degradation, (d) effects of different quenchers on the degradation efficiency of TC, the inset figures show the corresponding kinetic constants, (e) effect of the presence of N_2_ on TC degradation, conditions: [TC]_0_ = 10 μM, [PAA]_0_ = 100 μM, [BNQDs-FCN]_0_ = 0.2 g/L, [pH]_0_ = 7 ± 0.2, [TBA]_0_ = [MeOH]_0_ = [FFA]_0_ = 100 mM, [DMSO]_0_ = 40 mM, (f) the PMSO loss and PMSO_2_ production in the BNQDs-FCN/PAA system, [PMSO]_0_ = 120 μM, (g) EPR results of PAA, BNQDs-FCN and BNQDs-FCN/PAA with DMPO, (h) cyclic voltammetry curves, and (i) EIS Nyquist plots of CN, FCN, and BNQDs-FCN, The inset is an enlarged image.

To evaluate the degradation efficiency of the BNQDs-FCN/PAA system, TC was selected as the target antibiotic. As shown in Figure S2a, the system achieved a 91.70% degradation efficiency for TC. In contrast, when only BNQDs-FCN were present, the removal efficiency was only 8.38% after 30 min, indicating a negligible adsorption effect (Figure 3a). With the incorporation of Fe and BNQDs, the catalytic performance of CN was greatly promoted, increasing the degradation efficiency from 17.08% to 91.70%. The BNQDs-FCN/PAA system has the highest reaction rate constant (0.0843 min^–1^) and the highest PAA decomposition rate (78.36%) (Figure 3b). The rate constant was 21.6 times larger than that of the CN/PAA system.

The effect of the BNQDs-FCN dosage on TC degradation is shown in Figure S2b. Increasing the initial dosage from 0.025 to 0.2 g/L resulted in an improvement in TC degradation efficiency from 34.02% to 91.70% due to more reaction sites available for PAA activation. However, further increasing the dosage to 0.3 g/L showed a faster initial degradation rate, but the final efficiency after 30 min was nearly the same as that with 0.2 g/L. This shows that although the initial reaction rate was faster due to the greater number of active sites, the utilization of reactive surface sites was maximized at a dosage of 0.2 g/L. In addition, increasing the initial PAA concentration from 25 to 100 μM increased TC degradation efficiency by 20.41% and raised the degradation rate constant by 0.0437 min^–1^ (Figure S2c). However, further increasing the PAA dose to 150 and 200 μM suppressed the degradation efficiency, possibly because excess PAA scavenging free radicals.^37^ The effect of solution pH on the degradation efficiency of the BNQDs-FCN system is presented in Figure 3c. The catalyst’s isoelectric point is determined to be ∼6.4, indicating that its surface carries a negative charge under alkaline conditions (Figure S2d). With PAA’s dissociation constant (Ka) being ∼8.2, PAA mainly exists as its conjugated base PAA (PAA^–^) under strongly alkaline conditions, leading to electrostatic repulsion between the negatively charged catalyst and PAA, which reduces degradation efficiency.^38^ Additionally, the presence of OH^–^ can form complexes with Fe, resulting in precipitates that further decrease the catalytic activity of BNQDs-FCN. PAA is also more stable and difficult to decompose under acidic conditions.^39^ TC, an amphoteric antibiotic, exists as TCH_3_^+^ at pH values below 3.3 and as TCH^–^/TCH_2_^–^ at pH values above 7.7.^40^ Based on the catalyst’s isoelectric point, it can be inferred that at these pH extremes, the surface charges of the catalyst and TC are similar, leading to electrostatic repulsion, which hinders the advanced oxidation process on the catalyst surface. These factors indicate that the BNQDs-FCN/PAA system is most effective for treating wastewater under neutral pH conditions.

Quenching experiments were performed to discover the most important reactive species formed in the BNQDs-FCN/PAA catalyst (Figure 3d). *tert*-Butanol (TBA) was employed as a standard •OH quencher, while methanol (MeOH) could simultaneously quench •OH and organic radicals (R-O•: CH_3_C(O)O• and CH_3_C(O)OO•). Thus, the two quenchers could be used to dissociate the respective contributions of •OH and R-O•.^41,42^ When TBA and MeOH were present, the TC degradation efficiency decreased by 1.22% and 22.28%, respectively, indicating that R-O• and •OH were produced in the system, but •OH contributed negligibly to the degradation of TC. In addition, benzoic acid (BA) was used as a probe to further evaluate the contribution of •OH. Its sparse removal (6.16%) and low rate constant (0.002 min^–1^) confirmed the small contribution of •OH in the system (Figure S3).^42^ During the reaction, the peroxy-group formed by PAA decomposition could attack its central carbon atom to generate^1^O_2_,^43^ so an excess of furfuryl alcohol (FFA) was added to the reaction mixture to determine the contribution of^1^O_2_. After adding 100 mM of FFA, the removal rate of TC decreased by only 2.68%, indicating that^1^O_2_ was not the main reactive group for the degradation of TC. Because CH_3_• reacts rapidly with O_2_ to create CH_3_OO•, and CH_3_OO• has a significantly reduced oxidation capacity compared to CH_3_•, it is possible to determine the contributions of CH_3_OO• and CH_3_• by high purity N_2_ aeration.^44^ As shown in Figure 3e, the effect of N_2_ pass-through on TC decomposition is negligible, indicating that there was no formation of CH_3_OO• and CH_3_•. They did not participate in the degradation of TC.

Because the presence of Fe–N could result in the generation of high-valent iron-oxo species, dimethyl sulfoxide (DMSO) was selected to quench them. The addition of 40 mM DMSO reduced the degradation rate of TC by 63.04%, and the degradation rate constant was reduced from 0.0843 to 0.0105 min^–1^ (Figure 3d). These results implied that high-valent iron-oxo species are generated by the BNQOs-FCN/PAA complex, and they played a key role in the degradation of TC. Additionally, previous studies showed that high-valent iron-oxo species could oxidize methyl phenyl sulfoxide (PMSO) to methyl phenyl sulfone (PMSO_2_) via a mechanism involving the transfer of an oxygen atom, and thus, PMSO was used to chemically analyze the high-valent iron-oxo species.^45^ When PMSO was added to the BNQDs-FCN/PAA system, the concentration of PMSO decreased by 35.12 μM and the concentration of PMSO_2_ increased to 25.59 μM in 30 min (Figure 3f). The conversion efficiency of PMSO to PMSO_2_ was 65.68–72.88%, which indicated the generation of high-valent iron-oxo species.

An electron paramagnetic resonance (EPR) capture experiment was used to verify the composition of the reactive species. As shown in Figure 3g, neither BNQDs-FCN nor PAA has characteristic peaks. In the BNQDs-FCN/PAA system, a characteristic peak with an intensity ratio of 1:2:1:2:1:2:1 was detected. This signal indicated the rapid oxidation of DMPO to form DMPOX, confirming the presence of high-valent metal-oxo species in the BNQDs-FCN/PAA system.^46^ Previous studies confirmed that the rapid oxidation could be due to the co-oxidation of •OH, R-O•, and high-valent iron-oxo species.^47,48^ MeOH was added to the system to observe the change in the capture signal. Due to the existence of high-valent iron-oxo species, a strong oxidizing species, the peak shape of the generated signal did not change but the intensity decreased (Figure S4a). The standard 1:1:1 peak of TEMP–^1^O_2_ was also apparent, but a burst experiment proved that ^1^O_2_ did not serve a major role in the degradation of TC (Figure S4b). Therefore, free radicals (R-O• and •OH) as well as nonfree radicals (^1^O_2_ and high-valent iron-oxo species) were generated in the BNQDs-FCN/PAA system, with high-valent iron-oxo species playing a dominant role in the degradation of TC.

### Electron Transfer Mechanism

Figure 3h shows the cyclic voltammetry tests, where the reduction peak occurred at lower potentials and the oxidation peak occurred at higher potentials. The BNQDs-FCN system showed higher current peaks, indicating superior redox activity.^49^ In addition, Figure 3i shows the electrochemical impedance spectrum of BNQDs-FCN, which had the smallest semicircle diameter and possessed the best charge separation.^50^ Additionally, the semicircular ring diameters of FCN and CN were not large, but the change became especially obvious after doping with BNQDs, due to the high quantum confinement effect of BNQDs that enhanced the redox potential of the generated charges on the heterojunction catalyst.^51^

**Figure 4:**
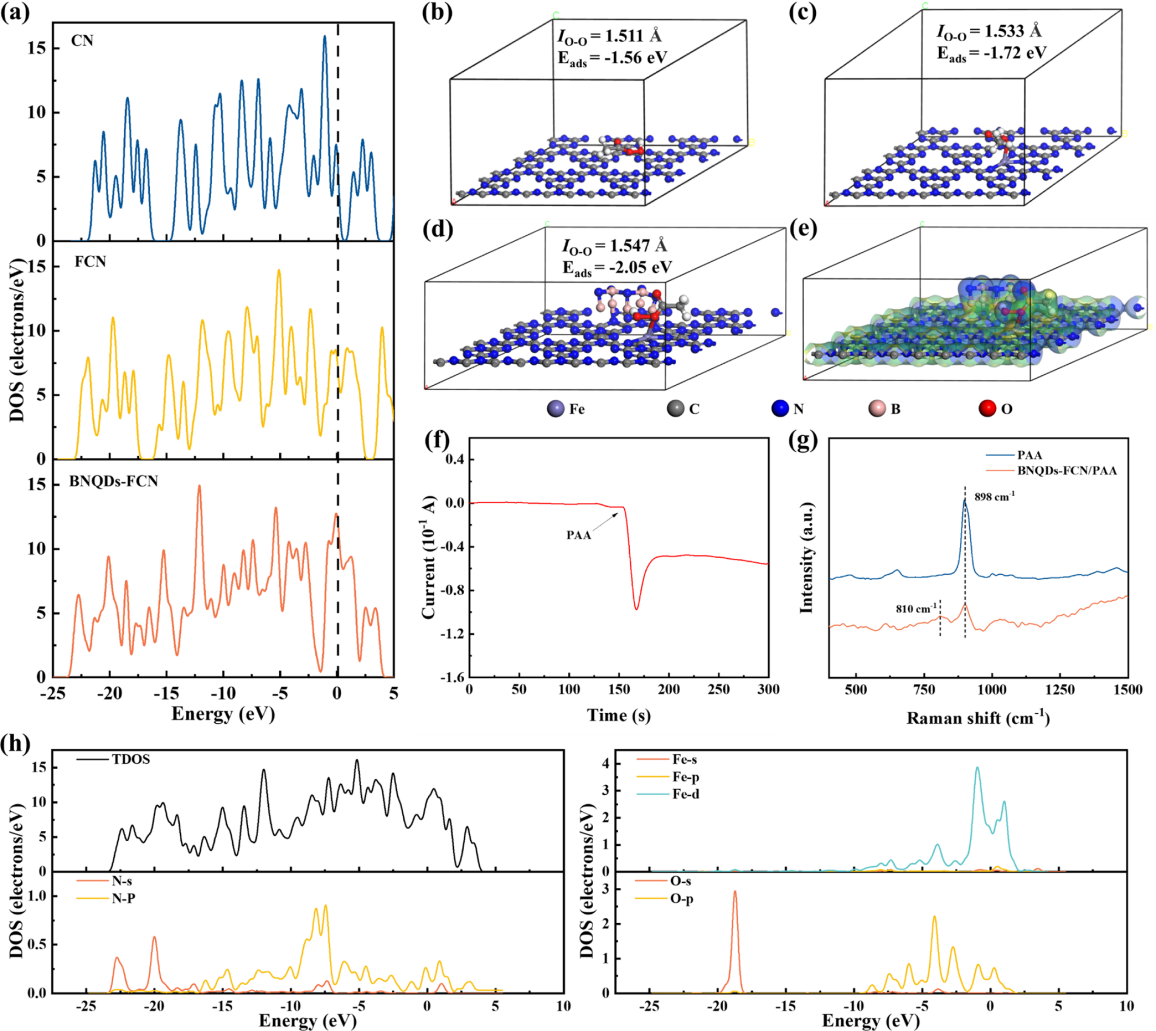
(a) DOS of CN, FCN, and BNQDs-FCN, adsorption energy of PAA on the surface of (b) CN, (c) FCN, and (d) BNQDs-FCN, (e) differential charge profile of PAA on BNQDs-FCN surface (the blue part: the electron accumulation region, the yellow part: the electron depletion region), (f) *in situ* Raman spectra of PAA and BNQDs-FCN/PAA, (g) current i-t curve after adding PAA. (h) DOS of BNQDs-FCN adsorbed PAA (TDOS: The total density of state).

The total densities of states (DOS) at the Fermi level for CN, FCN, and BNQDs-FCN are calculated to be 7.32, 8.37, and 12.78 electrons/eV, respectively (Figure 4a). The DOS near the Fermi level plays a critical role in electron transport within the material. Electrons must overcome the energy gap to transition between energy levels, and a higher DOS near the Fermi level means a greater concentration of free electrons, which leads to greater conductivity and more efficient charge transfer. Continuous material modifications increase the number of electrons near the Fermi energy level, further improving conductivity.^52^ These results indicate that BNQDs-FCN possesses greater charge transfer capacity, enhancing their ability to activate PAA.

The adsorption of PAA and the mechanism of advanced iron oxide generation were further analyzed by using DFT calculations. The adsorption energies (*E*_ads_) of PAA were calculated to evaluate the enhancement of PAA activation by Fe and BNQDs (Figure 4b–d). The *E*_ads_ values of PAA on CN, FCN, and BNQDs-FCN were −1.56, −1.72, and −2.05 eV, respectively, with more negative values indicating more energetically favorable adsorption. The results indicate that the binding affinity of the BNQDs-FCN structure was stronger, making PAA activation easier. Also, in the BNQDs-FCN/PAA system, the −O bond length of PAA was 1.547 Å, which was longer than those in CN (1.511 Å) and FCN (1.533 Å). The longer O–O bond was easier to break, which was favorable for the cleavage of PAA.

Additionally, charge density calculations for the BNQDs-FCN structure after PAA adsorption were used to examine the electron transfer mechanisms and the subsequent formation of the high-valent iron-oxo species. As shown in Figure 4e, the electron accumulation regions are shown in blue and the electron depletion region in yellow. The results revealed that Fe^3+^, acting as an electron donor, transfers electrons to PAA, leading to the generation of high-valent iron-oxo species and activation of PAA. In addition, the electron transfer pathway during the activation of PAA by BNQDs-FCN was further verified by chronoamperometry. It can be observed that when PAA was added, a negative current appeared, which also indicated that electrons were transferred from BNQDs-FCN to PAA (Figure 4f). At the same time, *in situ* Raman analysis is used to further verify the production of high-valent metal-oxo species in the system. As shown in Figure 4g, the characteristic peak at 898 cm^–1^ belongs to PAA.^53^ After the addition of the catalyst, the characteristic peak of PAA was weakened first, which also proved that it was activated and decomposed. A new peak was found at 810 cm^–1^, which was attributed to the production of high-valent metal-oxo species.^54^Figure 4f shows the DOS of the structure after the adsorption of PAA by BNQDs-FCN. The overlapping Fe-3d and *N*-2p peaks near the Fermi energy level confirm the high reactivity of the BNQDs-FCN structure. In addition, significant interaction between the Fe-3d, *N*-2p, and the O-2p orbitals indicates the activation of PAA through the elongation of the O–O bond. Also, the O-2p orbitals interact with Fe-3d to form a hybridized state, indicating the activation of PAA.

Meanwhile, an adsorption model for the pollutant TC on the catalyst was constructed with the adsorption energy calculated at −2.15 eV (Figure S4c). On the BNQDs-FCN catalyst, the central Fe atoms serve as both adsorption sites and reaction sites. Based on these collected results, an oxidation mechanism for the BNQDs-FCN/PAA system can be proposed (Figure S7). First, PAA adsorbs onto BNQDs-FCN, which leads to bond cleavage and activation of PAA with the help of the Fe–N site as an electron acceptor, while the system spontaneously generates high-valent iron-oxo species. Fe(II) in the lower valence state is converted to Fe(III) by electron transfer to produce CH_3_C(O)O• and CH_3_C(O)OO• simultaneously with PAA, facilitating the subsequent cyclic process. Additionally, the peroxy-group produced by the dissociation of PAA attacks the central C atom to produce^1^O_2_.

### Practicality and Applicability of the BNQDs-FCN/PAA System

**Figure 5:**
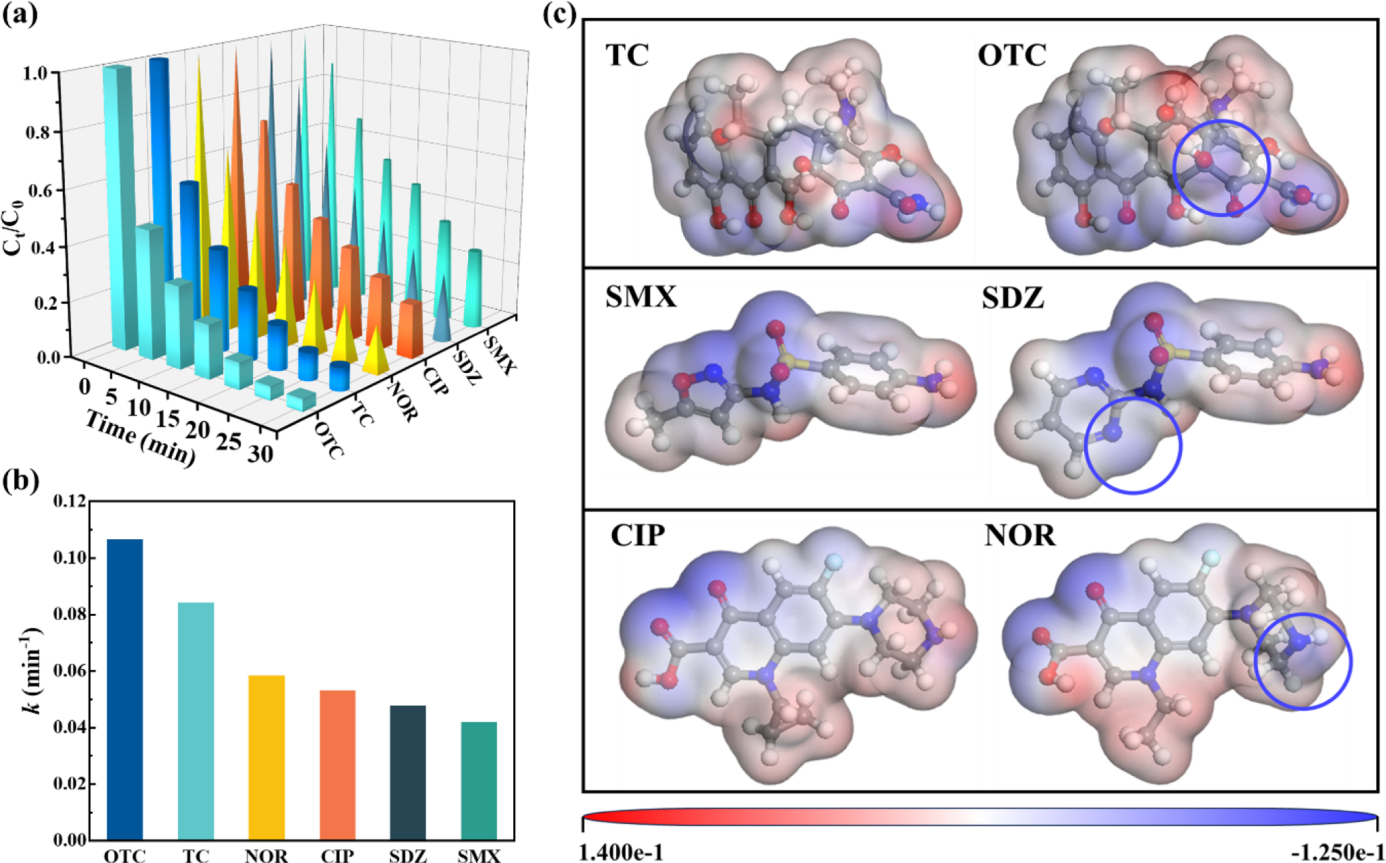
(a) Degradation of different antibiotics by the BNQDs-FCN/PAA process, (b) degradation rate of different antibiotics, conditions: [pollutants]_0_ = 10 μM, [PAA]_0_ = 100 μM, [BNQDs-FCN]_0_ = 0.2 g/L, [pH]_0_ = 7 ± 0.2, (c) electrostatic potential plots of different antibiotics (the red part: positive potential, the blue part, negative potential).

The effects of common anions and humic acids in water on the degradation process are shown in Text S6. The ability of the BNQDs-FCN/PAA system to decompose a variety of antibiotics, including TC antibiotics (such as oxytetracycline (OTC)), sulfonamide antibiotics (such as sulfadiazine (SDZ) and SMX), and quinolone antibiotics (such as norfloxacin (NOR) and ciprofloxacin (CIP)), was evaluated. The degradation rates of the aqueous materials of the OTC, NOR, CIP, SDZ, and SMX after 30 min were 95.57%, 83.16%, 80.53%, 75.29%, and 71.38%, respectively (Figure 5a). With the extended treatment, the degradation efficiency of all pollutants exceeded 80% (Figure S8). Among them, OTC, SDZ, and NOR are relatively easier to degrade and have higher degradation rate constants. Specifically, OTC‘s rate constant was 0.0222 min^–1^ higher than TC, SDZ’s was 0.0031 min^–1^ higher than SMX, and NOR’s was 0.0058 min^–1^ higher than CIP (Figure 5b).

DFT calculations were employed to assess changes in the surface-active sites of the antibiotics due to their different structures, which could help explain the selective attack by high-valent iron-oxo species. As shown in Figures S9 and S14, the Fukui indices of the six antibiotic molecules were calculated. The Fukui index, which measures the differential change in electron density in specific regions of molecules when the number of electrons or the total charge varies under a constant external potential, effectively predicts the reactivity of different regions. The Fukui (−) values indicate changes in electron density following the loss of electrons from the natural bonding orbital, suggesting an electrophilic reaction. The Fukui (−) value was larger for the outermost oxygen atom of the structure of each antibiotic (indicated by the yellow areas), which indicated that electrophilic attack was prone to occur at this site. As a positively charged and strongly oxidizing substance, the high-valent iron-oxo species are highly electrophilic; thus, they could easily attack this oxygen site.

Considering other antibiotics, it is anticipated that during degradation in the BNQDs-FCN/PAA system, the generated high-valent iron-oxo species were the first to attack oxygen-containing groups such as hydroxyls, carbonyls, and S=O bonds, which would damage the antibiotic from the outside of the structure. In addition, Fukui (+) reflects the ability of a certain point in a molecule to accept electrons, indicating its susceptibility to nucleophilic attack, while Fukui (0) can measure the molecule’s vulnerability to attack by free radicals. In this system, R-O• is produced as a free radical that targets sites with large Fukui (0) values. Therefore, taking TC as an example, the degradation process of TC in the BNQDs-FCN/PAA system, as well as the toxicity of its degradation products, were studied in Text S7. The Fukui function was used to identify the vulnerable regions of the contaminant based on local atomic interactions.

Furthermore, the sites in the molecular structure that are susceptible to high-valent iron oxide species were further analyzed in terms of the whole by means of electrostatic potential (ESP). The electrostatic potential is a physical quantity that describes the distribution of potential energy in an electric field and directly reflects the force acting on a unit positive charge at each point in space. The electrostatic potential at each point in an electrostatic field can be interpreted as a measure of potential energy, i.e., the work required to move a unit positive charge from infinity to that point. The positive or negative value of this physical quantity indicates the nature of the repulsion or attraction for a positive charge. Understanding the distribution of the electrostatic potential around the molecular structure is also particularly important in the study of the effects of high-valent iron oxides on pollutants. The electrostatic potential, which can reveal the distribution of charges between different molecules as well as within molecules, was calculated for the antibiotic molecular structure. Regions of positive electrostatic potentials indicate that the molecule is repulsive to positive charges at that site, while negative electrostatic potentials indicate attraction. To show the variation of the surface-active sites, the electrostatic potential of the antibiotic structure was plotted as shown in Figure 5c, with negatively charged groups indicated in blue and positively charged groups in red. These negatively charged parts were easily attacked by the high-valent iron-oxo species. Examination of different substances of the same broad class of antibiotics revealed that the portions of OTC, SDZ, and NOR were more negatively charged than those of TC, SMX, and CIP, respectively (blue circle). The areas highlighted in green in Figures S9–S14 indicate their respective atoms also had larger Fukui (−) values, which could be due to structural differences. This analysis revealed that OTC, SDZ, and NOR contain more regions susceptible to attack by high-valent iron-oxo species, making them more prone to degradation compared with other antibiotics in the same class, which aligns with the findings in Figure 5a.

### Performance Comparison and Reactor Design

The pollutant degradation levels of different Fe–N catalysts were compared for their treatment performance of the systems, and the BNQDs-FCN/PAA system demonstrated an excellent degradation rate constant (0.0843 min^–1^) (Table S3). Based on this experimental study, a preliminary design of a water treatment reactor is proposed for industrialization. As shown in Figure S18, wastewater is introduced into the bottom of the reaction tower through a peristaltic pump, flowing upward. Catalysts and oxidants are added above the tower to create a countercurrent flow, enhancing the degradation efficiency. An aeration device at the bottom of the tower increases the interactions between catalyst particles and pollutants, facilitating better contact and separation, exposing more reaction sites, and accelerating the degradation process. The treated wastewater is then discharged through another peristaltic pump. This setup is designed for wastewater treatment, effectively degrading persistent antibiotic pollutants.

### Environmental Implications

The BNQDs-FCN catalyst, prepared through pyrolysis technology, shows significant potential in wastewater treatment by activating PAA. Studies indicate that PAA acts as an electron acceptor during activation, facilitating charge transfer within the central iron atom and forming high-valent metal-oxo species. This process significantly enhances the catalyst’s oxidation ability. The BNQDs-FCN/PAA system shows strong environmental stability in practical applications, especially in its resistance to interfering substances, such as common anions and HA. This feature enables it to maintain a high removal efficiency in complex water systems, especially when treating antibiotics such as TC, where the system achieves a satisfactory degradation rate.

This demonstrates the system’s effectiveness and provides a practical solution for degrading antibiotics. The presence of high-valent metal-oxo species can effectively attack oxygenated groups in antibiotic molecules, further improving the degradation performance. This feature is of great significance in practical applications, where water bodies contaminated by antibiotics are often complex and changeable, making single treatment methods insufficient. The BNQDs-FCN/PAA system not only achieves efficient TC degradation but also significantly reduces the toxicity of pollutants, thus contributing to environmental safety and reducing ecological risks.

In addition to its technical feasibility, this system offers economic and environmental benefits. PAA, as an organic acid, may also serve as a carbon source for microbial growth, suggesting that the combination of PAA-AOPs and biological treatment technology could further improve the overall efficiency of wastewater treatment. In summary, the BNQDs-FCN/PAA system demonstrates strong potential for treating organic pollutants, showcasing its feasibility, cost-effectiveness, and practical application value. This study provides innovative insights for future environmental protection technologies and is expected to contribute positively to water pollution mitigation and ecological preservation.

## Supporting Information Available

Details of reagents, catalyst preparation, experimental details, analytical methods, and DFT calculation methods (PDF)
