# Supplementary material for: Activation of PAA at the Fe–Nx Sites by Boron Nitride Quantum Dots Enhanced Charge Transfer Generates High-Valent Metal-Oxo Species for Antibiotics Degradation
Source: Environ Sci Technol. 2024 Nov 28;58(49):21871–81. doi: 10.1021/acs.est.4c08224 (PMC11709145; doi:10.1021/acs.est.4c08224)
Supplement: Supplementary file 1 — es4c08224_si_001.pdf [file es4c08224_si_001.pdf]

Supporting Information

The activation of PAA at the Fe-Nx sites by boron nitride quantum dots enhanced charge transfer generates high-valent metal-oxo species for antibiotics degradation

Shuo Li[a], Yalun Yang[b], Junfeng Niu[b], Heshan Zheng[a], Wen Zhang[c], Yoong Kit Leong[d,e], and Jo-Shu Chang[d,e,f*], Bo Lai[g*]*

[a]College of Chemistry and Chemical Engineering, Qiqihar University, Qiqihar, 161006, China

[b]College of Environmental Science and Engineering, North China Electric Power University, Beijing 102206, China

[c]John A. Reif, Jr. Department of Civil and Environmental Engineering, New Jersey Institute of Technology, Newark, NJ 07102, USA

[d]Department of Chemical and Materials Engineering, Tunghai University, Taichung 407, Taiwan

[e]Research Center for Smart Sustainable Circular Economy, Tunghai University, Taichung 407, Taiwan

[f]Department of Chemical Engineering and Materials Science, Yuan Ze University, Chung-Li, Taiwan

[g]Department of Environmental Science and Engineering, School of Architecture and Environment, Sichuan University, Chengdu 610065, China

Summary

30 pages, 7 Texts, 18 Figures, 3 Tables.

Table of Contents

Text S1. Chemical reagents S4

Text S2. Catalyst preparation S4

Text S3. Experimental details S5

Text S4. Analytical methods S5

Text S5. DFT calculation methods S7

Text S6. Effects of water environmental disturbances on degradation processes. S7

Text S7. Degradation pathway analysis and intermediate product toxicity prediction. S8

Figure S1. (a) TEM of CN and FCN, (b) AFM images, the left is a plane view, and the right is a 3D view, (c) fluorescence image of BNQDS-FCN: reactive photovoltaic properties and charge transfer capability XPS high-resolution spectrum of (d) Fe and (e) B of FCN and BNQDs-FCN, (f) FT-EXAFS fitting curve in R space of BNQDs-FCN, (g) K space spectra of the Fe K-edge of BNQDs-FCN. S10

Figure S2. (a) Effect of Fe and BNQDs content on the degradation of TC, (b) effect of initial dosage of catalyst on TC degradation, (c) effect of PAA initial concentration on TC degradation, the inset figures show the corresponding kinetic constants, conditions: $[TC]_0 = 10$ μM, $[PAA]_0 = 100$ μM, $[BNQDs\text{-}FCN]_0 = 0.2$ g/L, $[pH]_0 = 7 \pm 0.2$, (d)zeta potential and the IEP of BNQDs-FCN. S11

Figure S3. BA degradation by the BNQDs-FCN/PAA process, conditions: $[BA]_0 =10$ μM, $[PAA]_0 = 100$ μM, $[BNQDs\text{-}FCN]_0 = 0.2$ g/L, $[pH]_0 = 7 \pm 0.2$. S12

Figure S4. (a) EPR results of BNQDs-FCN/PAA with DMPO, (b) EPR results of PAA alone and BNQDs-FCN/PAA with TEMP, (c) adsorption energy of TC on the surface of BNQDs-FCN. S13

Figure S5. The effect of different water matrices on the degradation of TC: (a) CO_3^{2-}, (b) HCO_3^{2-}, (c) NO_3^-, (d) Cl- (e) HA, (f) effects of different water bodies, the inset figures show the corresponding kinetic constants, conditions: $[TC]_0 = 10$ μM, $[PAA]_0 = 100$ μM, $[BNQDs\text{-}FCN]_0 = 0.2$ g/L, $[pH]_0 = 7 \pm 0.2$.S14

Figure S6. (a) Reusability of catalyst for the degradation of TC and metal ion leaching concentration during recycling experiments, (b) Effects of leached metals on TCH degradation, conditions: $[TC]_0= 10$ μM, $[PAA]_0= 100$ μM, $[Fe]_0 = 0.2$ g/L, $[pH]_0 = 7 \pm 0.2$ S15

Figure S7. The Fukui index of atoms in NOR molecule. S16

Figure S8. Degradation rates of SDZ and SMX after 30min, conditions: $[TC]_0= 10$ μM, $[PAA]_0= 100$ μM, $[BNQDs\text{-}FCN]_0 = 0.2$ g/L, $[pH]_0 = 7 \pm 0.2$. S17

Figure S9. The Fukui index of atoms in TC molecule. S18

Figure S10. The Fukui index of atoms in OTC molecule S19

Figure S11. The Fukui index of atoms in SMX molecule. S20

Figure S12. The Fukui index of atoms in SDZ molecule. S21

Figure S13. The Fukui index of atoms in CIP molecule. S22

Figure S14. The Fukui index of atoms in NOR molecule. S23

Figure S15. Mass spectra of intermediates at different reaction times: (a) 0 min, (b) 15 min, and (c) 30 min. S24

Figure S16. Degradation pathways of TC. S25

Figure S17. Change of TOC in the BNQDs-FCN/PAA system, conditions: $[TC]_0 = 10$ μM, $[PAA]_0 = 100$ μM, $[BNQDs\text{-}FCN]_0 = 0.2$ g/L, $[pH]_0 = 7 \pm 0.2$. S26

Figure S18. Process flow chart. S27

Table S1. EXAFS fitting parameters at the Fe K-edge for BNQDs-FCN. S28

Table S2. Prediction of acute and chronic toxicity of TC and intermediates in the BNQDs-FCN/PAA systems by ECOSAR. S28

Table S3. Comparison of pollutant degradation levels by different Fe-N catalysts. S28

References..S30

Text S1. Chemical reagents

Tetracycline (TC), oxytetracycline (OTC), sulfadiazine (SDZ), sulfamethoxazole (SMX), norfloxacin (NOR), ciprofloxacin (CIP), humic substance (HA), 2,2,6,6-tetramethyl-1-piperidinyloxy (TEMP), 5,5-dimethyl-1-pyrroline N-oxide (DMPO), N,N-diethyl-p-phenylenediamine (DPD), peracetic acid (PAA, 15%-18%), p-benzoquinone (p-BQ), furfuryl alcohol (FFA), acetonitrile (HPLC grade), methyl phenyl sulfoxide (PMSO), methyl phenyl sulfone ($PMSO_2$), hexagonal boron nitride (h-BN), and ferric chloride ($FeCl_3$) were purchased from Aladdin Co., Ltd. (Shanghai, China), hydrogen peroxide (H_2O_2, 30%), sodium hydroxide (NaOH), sodium chloride (NaCl), sodium carbonate (Na_2CO_3), tert-butanol (TBA), and methanol (MeOH) were purchased from Sinopharm Chemical Reagent Co., Ltd. (Shanghai, China). Melamine, formic acid (HPLC grade), and acetic acid (HPLC grade) were purchased from Kermel Chemical Reagent Co., Ltd. (Tianjin, China).

Text S2. Catalyst preparation

BNQDs were prepared using the top-down liquid-phase ultrasound stripping method. First, 0.3 g of h-BN was dissolved in 30 mL of water, and the mixed solution was subsequently placed in an ultrasonic cell disintegrator for 4 h (working 1 s at 2 s intervals) and then 5 h (working 2 s at 1 s intervals) in an ice bath sonication process. The suspensions were then sealed and sonicated using a water bath sonication unit at less than 10 ^oC for 48 h. Finally, the suspensions were centrifuged at 10,000 rpm for 10 min, and the supernatants were collected to obtain BNQDs. A certain amount of $FeCl_{3,}$ BNQDs, and 5 g of melamine were dissolved in 50 mL of water and stirred at 400 rpm for 24 h. Subsequently, the mixed solution was placed in a desiccator, and the solvent was evaporated at 60 ^oC. The resulting solid was ground, sieved, and subsequently transferred to a muffle furnace, which ramped up to 550 ^oC at 3 ^oC/min and maintained under a self-sustaining atmosphere for 4 h. The product was washed with deionized water to remove impurities, followed by drying, grinding, and sieving to obtain BNQDs-FCN. CN and FCN were prepared under the same preparation conditions without adding Fe and BNQDs, respectively. Depending on the dose of $FeCl_3$ added (0.025, 0.05, 0.075, and 0.1 M), the resulting products were denoted

as FCN1, FCN2, FCN3, and FCN, and depending on the dose of BNQDs added (2, 4, 6, 8, and 10 mL), the resulting products were denoted as 2BNQDs-FCN, 4BNQDs-FCN, 6BNQDs-FCN, 8BNQDs-FCN, and BNQDs-FCN, respectively.

Text S3. Experimental details

Degradation experiments were carried out in a 250 mL beaker containing 100 mL of contaminant solution with a predetermined concentration of PAA, and the initial pH of the reaction was adjusted with 0.1 M NaOH and H_2SO_4, followed by the addition of a predetermined amount of catalyst to initiate the reaction. During the experimental procedure, the temperature was room temperature (25 ± 2 ^oC) and the magnetic stirring speed was 400 rpm. Samples were collected at predetermined time intervals and filtered through a 0.22 μm filter membrane, after which the reaction was terminated by adding excess sodium thiosulfate immediately. The samples were stored at 4 ^oC for further analytical testing. In the degradation experiment of different water bodies, TC solution was prepared with tap water from the Qiqihar water plant and surface water from Labor Lake.

Text S4. Analytical methods

PAA solutions were diluted from stock solutions and periodically calibrated by two-step titrations. The concentration of hydrogen peroxide in the solution was determined by iodometric measurement, followed by titration of H_2O_2 with potassium permanganate at acidic pH. The PAA concentration was the concentration of hydrogen peroxide minus the concentration of H_2O_2. The concentration of residual PAA in the reaction was determined spectrophotometrically using N, N-diethyl-p-phenylenediamine (DPD). The concentration of TC was detected by high-performance liquid chromatography (HPLC, Nexera LC-40, Shimadzu, Japan) equipped with a Shim-pack GIST C18 column (2.1×50 mm, 2μm) and a UV detector, with acetonitrile and 0.1% formic acid in the ratio of 30:70 as mobile phase, the flow rate was 0.1 mL/min, the temperature of the column oven was 30 ^oC and the detection wavelength was 360 nm. For the detection of OTC, the mobile phase was acetonitrile and 0.1% formic acid in the ratio of 30:70, the flow rate was

0.1 mL/min, the column temperature was 30 °C, and the detection wavelength was 355 nm. For the detection of SMX, the mobile phase was acetonitrile and ultrapure water in the ratio of 30:70, the flow rate was 0.2 mL/min, the column temperature was 35 °C, and the detection wavelength was 265 nm. For the detection of SDZ, the mobile phase was acetonitrile and ultrapure water in the ratio of 25:75, the flow rate was 0.2 mL/min, the column temperature was 35 °C, and the detection wavelength was 270 nm. For the detection of CIP, the mobile phase was acetonitrile and 0.2% formic acid aqueous solution in the ratio of 20:80, the flow rate was 0.3 mL/min, the column temperature was 30 °C, and the detection wavelength was 277 nm. For the detection of NOR, the mobile phase was acetonitrile and 0.2% formic acid aqueous solution in the ratio of 20:80, the flow rate was 0.3 mL/min, the column temperature was 30 °C, and the detection wavelength was 272 nm. For the detection of PMSO, the mobile phase was acetonitrile and 0.1% formic acid aqueous solution in the ratio of 15:85, the flow rate was 0.5 mL/min, the column temperature was 30 °C, and the detection wavelength was 230 nm. For the detection of PMSO2, the mobile phase was acetonitrile and 0.1% formic acid aqueous solution in the ratio of 25:75, the flow rate was 0.5 mL/min, the column temperature was 30 °C, and the detection wavelength was 215 nm. All injection volumes were 5 μL. The concentration of metal ions was determined by inductively coupled plasma mass spectrometry (ICP-MS, NexION350X, Perkin Elmer, USA). The free radicals generated in the system solution were detected by electron paramagnetic resonance (EPR, A300E, Bruker, Germany). Degradation intermediates of TC were analyzed by ultra-high liquid chromatography-mass spectrometry, using acetonitrile and 0.1% formic acid as the mobile phase. The ESI + mode voltage is 3.8 kV, the gas flow rate is 15 $Lmin^{-1}$, and the gas temperature is 150 °C. The toxicity of the degraded TC intermediates was analyzed using the quantitative constitutive relationship (QSAR)--based ecological constitutive relationship modeling (ECOSAR) software. The chemical reaction rate refers to the rate at which the concentration of reactants decreases, and the concentration of products increases per unit time, serving as a measure of the reaction's speed. The quantitative relationship that describes how the reaction rate is influenced by the amount or concentration of the substances participating in the reaction is known as the chemical reaction rate equation, with its general form shown in Eq. (1). In current research on the advanced oxidation degradation

of pollutants, first-order reactions are the most commonly observed, with their kinetic equation shown in Eq. (2). First, the concentration of TC at time t is detected by liquid chromatography to obtain the value of ln (C_t/C_0). Then, a graph of the relationship between ln (C_t/C_0) and time t is plotted, and finally, the slope (-k) is obtained.

$$\frac{dC}{dt} = - kC^n \quad (1)$$

$$\ln(\frac{C_t}{C_0}) = kt \quad (2)$$

C is the concentration of the reactant (mg/L), t is the reaction time (min), C_t is the concentration of the reactant at time t (mg/L), C_0 is the initial concentration of the reactant (mg/L), *k* is the chemical reaction kinetic rate constant (min^{-1}), n is the reaction order.

Text S5. DFT calculation methods

All DFT calculations were performed based on the Materials Studio (MS) software. The catalyst structure was optimized using the Perdew-Burke-Ernzerhof (PBE) functional of the generalized gradient approximation in the CASTEP module. The energy and force convergence were set to 1.0×10^{-4} eV and 0.02 eV $Å^{-1}$, respectively. Then, the density of states (DOS) of the optimized catalyst was calculated to study the electronic structure of the catalyst. The energy cutoff for the plane wave unfolding was set to 500 eV, and a vacuum space of 15 Å was used above the surface to avoid periodic interactions. Meanwhile, the energy of the catalyst substrate, PAA, and PAA adsorbed substrate was calculated to obtain the adsorption energy of PAA ($E_{ads} = E_{sub*PAA}-E_{PAA}-E_{sub}$). In addition, to understand the possible degradation sites of various antibiotics at the level of theoretical calculations, the Dmol3 module was used to optimize the structures of various antibiotic molecules and calculate the electrostatic potential and the Fukui function.

Text S6. Effects of water environmental disturbances on degradation processes.

Common anions were added to assess the real-world applicability of the BNQDs-FCN/PAA system. In the presence of 10 mM CO_3^{2-}, HCO_3^{2-}, NO_3^-, and Cl^-, the degradation rate of TC was only inhibited by

8.5%, 3.4%, 1.44%, and 7.67%, respectively (Figure S5a-d). These results demonstrate that the strong oxidizing properties of the high-valent iron-oxo species improved the system's resistance to interference from other chemicals. The degradation rate of TC decreased to 65.15% after the addition of 10 mg/L of humic substance (HA) to the system because HA could occupy the reactive sites on the catalyst surface, resulting in the reduction of the number of active species produced by the system, and it also competes with TC for the active species (Figure S5e). In addition, the ability of the system to decompose TC in surface water (SW) and tap water (TW) was evaluated, in which the degradation rates of TC after 30 min decreased by 33.19% and 11.23%, respectively (Figure S5f). The greater decrease in SW could be due to the competition of soluble organic matter present in the water for the active species. The reusability of a catalyst is one of its most important properties for real-world applications. The removal efficiency of TC was tested using recycled BNQDs-FCN, and the system still achieved a degradation rate of 82.6% after five cycles, which reflects the stable nature of the catalyst and its recyclability. The amount of leached Fe was also determined, and by the end of the fifth cycle, it had increased to 0.1075 mg/L (Figure S6a), which is well below the limit set by the Chinese drinking water hygiene standard (GB 5749-2022, Fe: 0.3 mg/L). In addition, it was found that the effect of Fe leaching on TC degradation was negligible (Figure S6b).

Text S7. Degradation pathway analysis and intermediate product toxicity prediction.

High-performance liquid chromatography-mass spectrometry (HPLC-MS) was utilized to determine the degradation intermediates of the TC solutions after different degradation reaction times (Figure S15). The degradation pathways that generated these intermediates were determined in conjunction with DFT calculations. As shown in Figure S16, two main oxidation pathways were proposed. First, TC generated TP-417 and TP-427 by demethylation and dehydroxylation, respectively. The external oxygen-containing groups with higher electron density were attacked by high-valent iron-oxo species, and the large Fukui (0) values at C16 and O23 were susceptible to the attack of R–O•. Therefore, TP-417 and TP 427 subsequently generated intermediates such as TP-317, TP-298, TP-274, TP-259, and TP-243, which are often accompanied by C–C and C–N bond cleavage, deamination, ring-opening reactions, hydroxylation, and

loss of functional groups. Hydroxylation could occur because of small amounts of •OH that were produced by the system. These intermediates then produced small molecule intermediates, such as TP-84, TP-90, and TP-146, through major ring-opening reactions. Finally, these small-molecule products were gradually mineralized to CO_2 and H_2O. The plan is to determine the degree of mineralization by detecting changes in TOC, but PAA itself is an organic peroxide, and its addition will affect the determination of TOC. As shown in Figure S17, the concentration of TOC in the system increases. In addition, the Ecological Structure-Activity Relationships Model, which is based on quantitative structure-activity relationships, was used to predict the toxicity of the intermediates formed in the degradation of TC by BNQDs-FCN/PAA (Table S2). The intermediates TP-84, TP-90, and TP-146 formed by the final transformation had higher acute and chronic toxicity values than TC, indicating that the ecological risk of TC was significantly reduced.

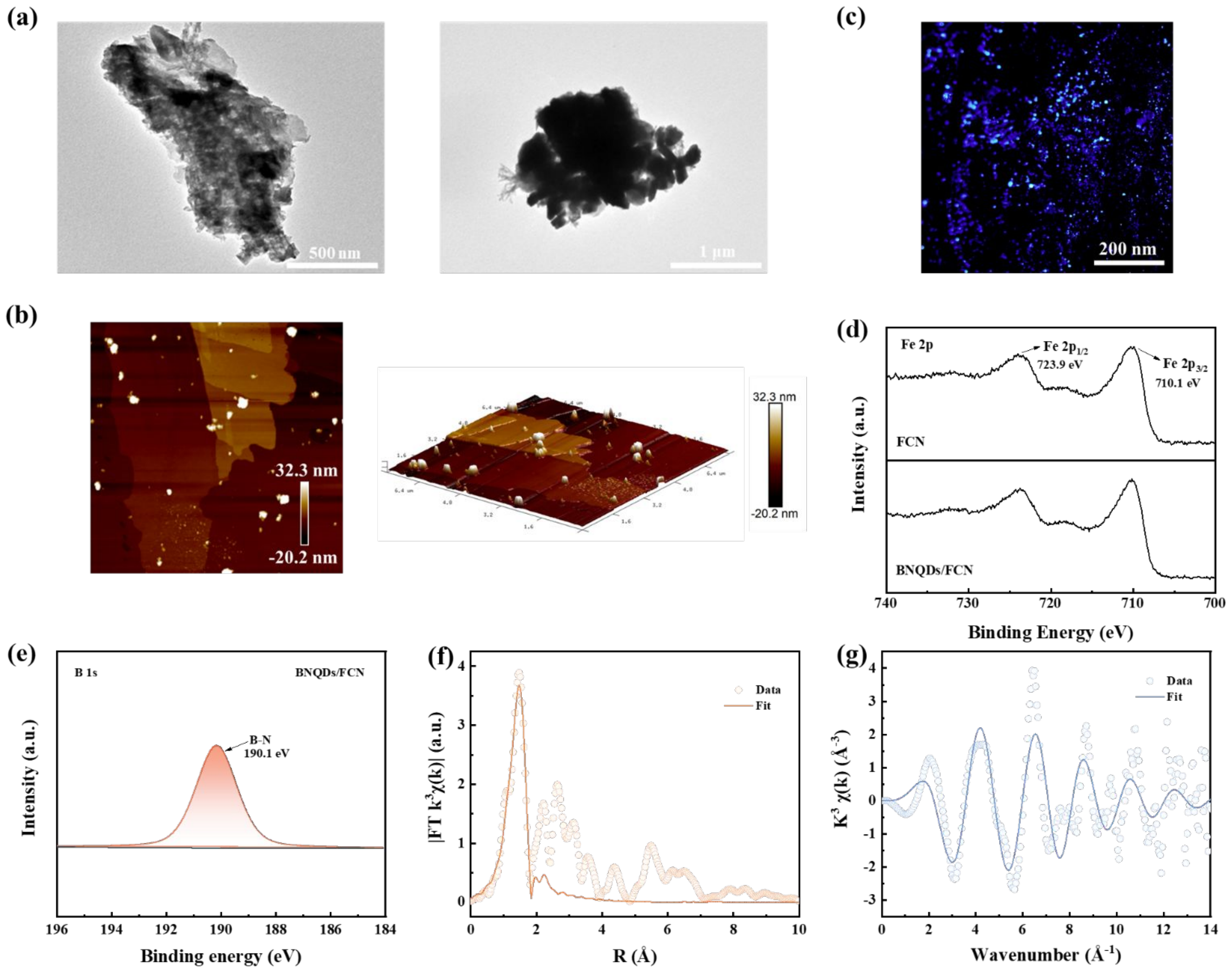

Figure S1. (a) TEM of CN and FCN, (b) AFM images, the left is a plane view, and the right is a 3D view, (c) fluorescence image of BNQDS-FCN: reactive photovoltaic properties and charge transfer capability XPS high-resolution spectrum of (d) Fe and (e) B of FCN and BNQDs-FCN, (f) FT-EXAFS fitting curve in R space of BNQDs-FCN, (g) K space spectra of the Fe K-edge of BNQDs-FCN.

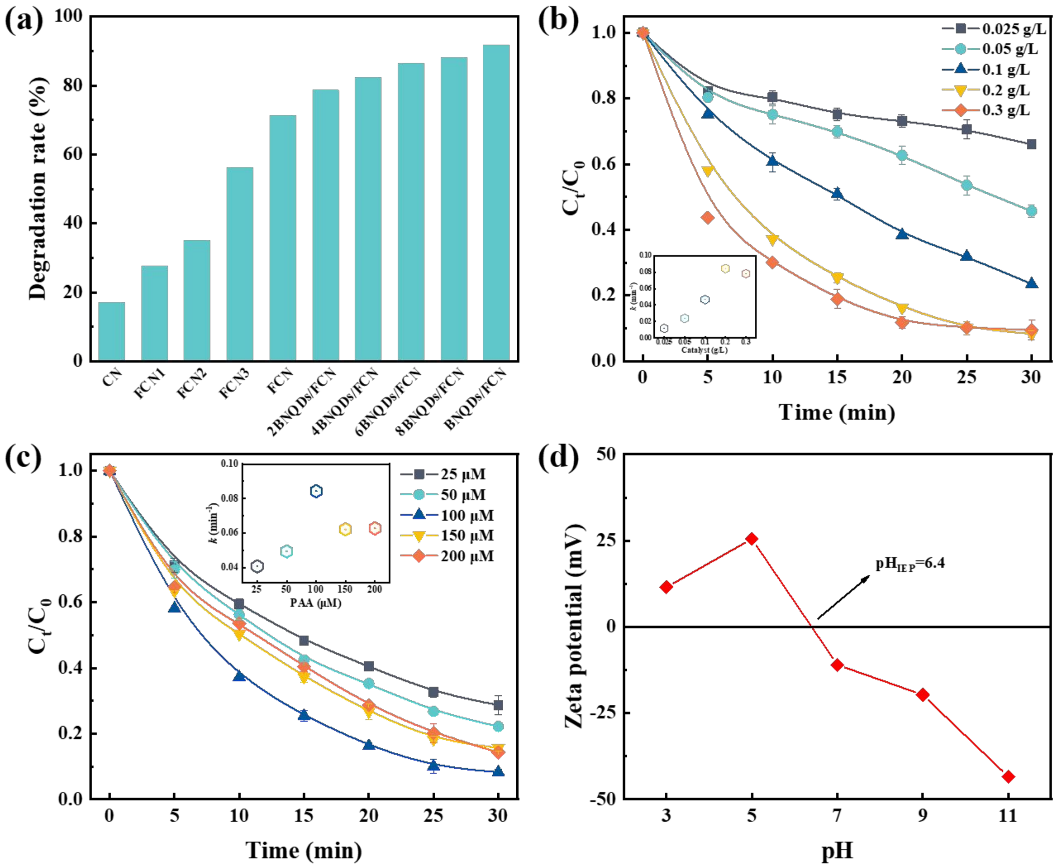

Figure S2. (a) Effect of Fe and BNQDs content on the degradation of TC, (b) effect of initial dosage of catalyst on TC degradation, (c) effect of PAA initial concentration on TC degradation, the inset figures show the corresponding kinetic constants, conditions: $[TC]_0 = 10$ μM, $[PAA]_0 = 100$ μM, $[BNQDs\text{-}FCN]_0 = 0.2$ g/L, $[pH]_0 = 7 \pm 0.2$, (d)zeta potential and the IEP of BNQDs-FCN.

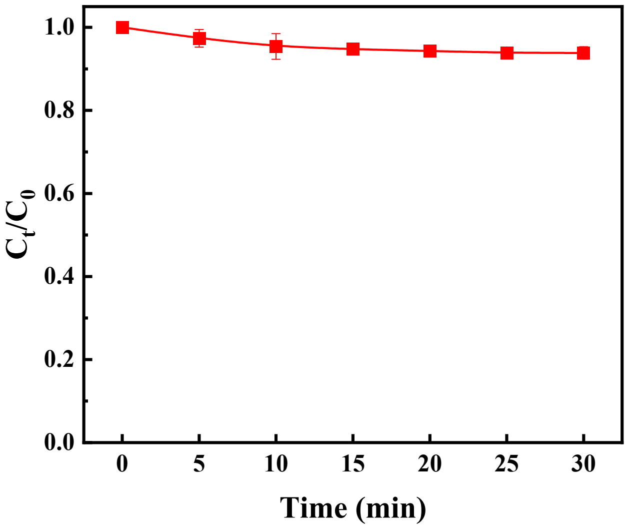

Figure S3. BA degradation by the BNQDs-FCN/PAA process, conditions: $[BA]_0$ =10 μM, $[PAA]_0$ = 100 μM, $[BNQDs\text{-}FCN]_0$ = 0.2 g/L, $[pH]_0$ = 7 ± 0.2.

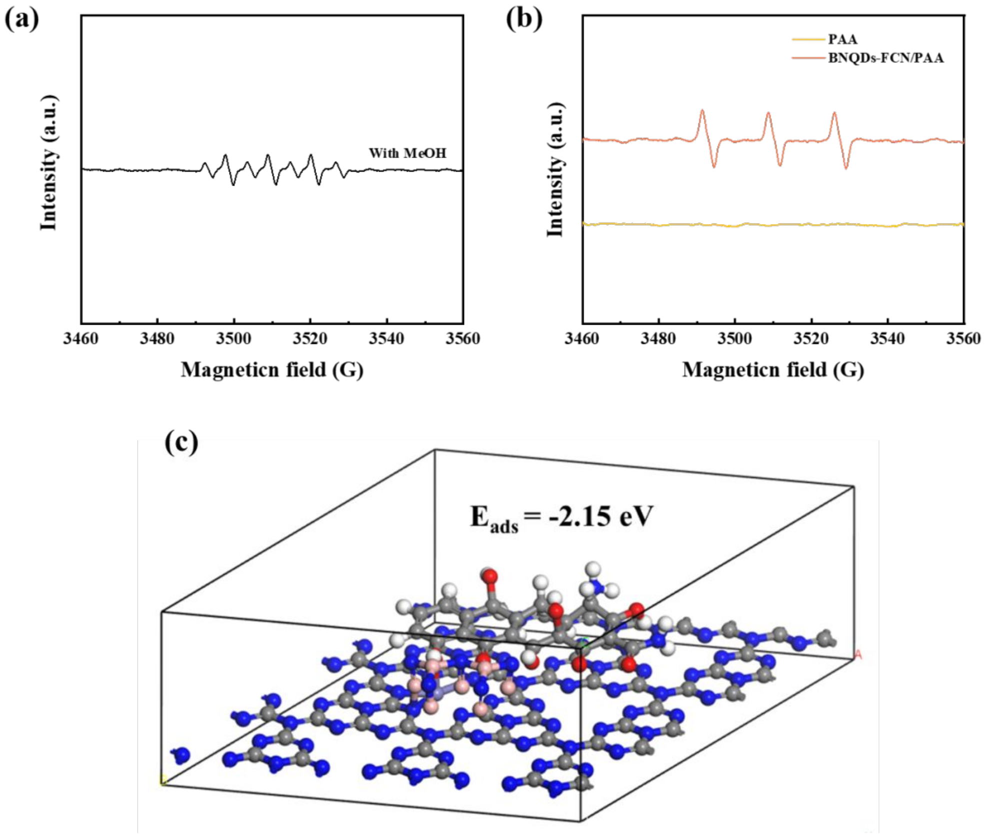

Figure S4. (a) EPR results of BNQDs-FCN/PAA with DMPO, (b) EPR results of PAA alone and BNQDs-FCN/PAA with TEMP, (c) adsorption energy of TC on the surface of BNQDs-FCN.

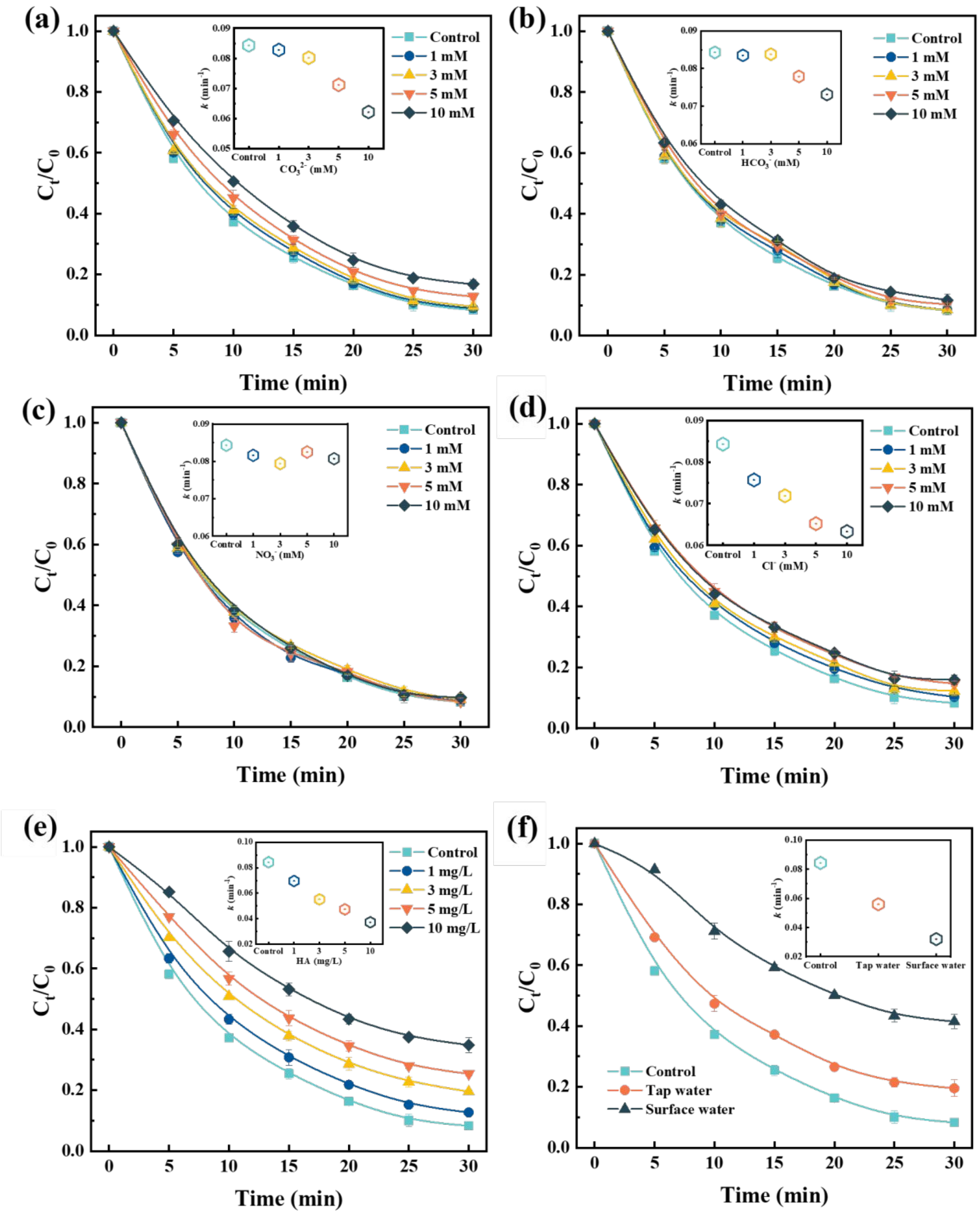

Figure S5. The effect of different water matrices on the degradation of TC: (a) CO_3^{2-}, (b) HCO_3^{2-}, (c) NO_3^-, (d) Cl- (e) HA, (f) effects of different water bodies, the inset figures show the corresponding kinetic constants, conditions: $[TC]_0 = 10$ μM, $[PAA]_0 = 100$ μM, $[BNQDs\text{-}FCN]_0 = 0.2$ g/L, $[pH]_0 = 7 \pm 0.2$.

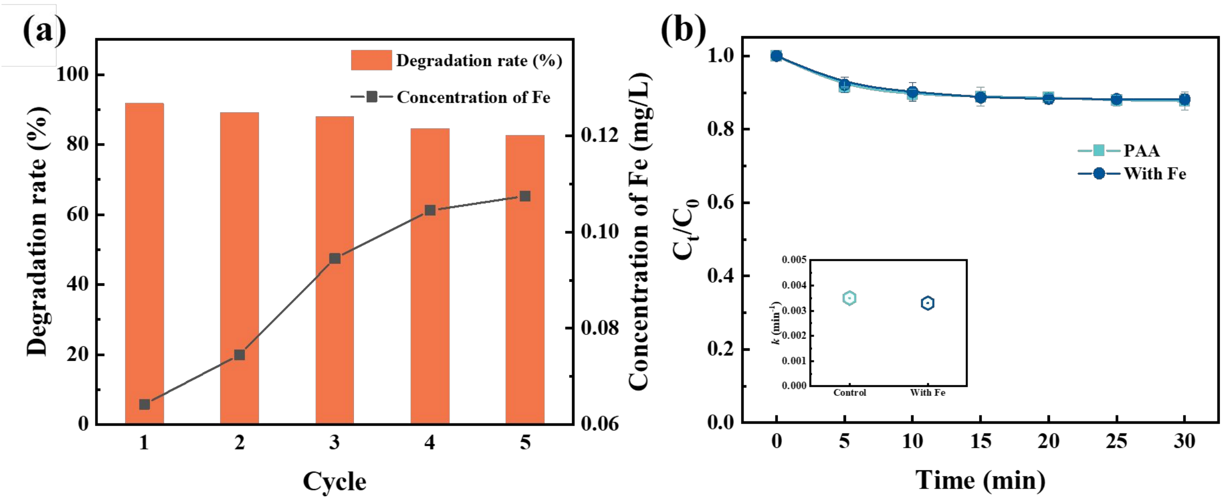

Figure S6. (a) Reusability of catalyst for the degradation of TC and metal ion leaching concentration during recycling experiments, (b) Effects of leached metals on TCH degradation, conditions: $[TC]_0$= 10 μM, $[PAA]_0$= 100 μM, $[Fe]_0$ = 0.2 g/L, $[pH]_0$ = 7 ± 0.2.

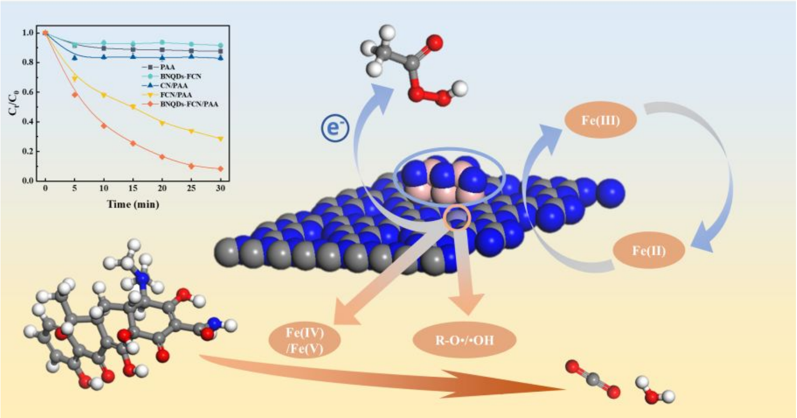

Figure S7. The Fukui index of atoms in NOR molecule.

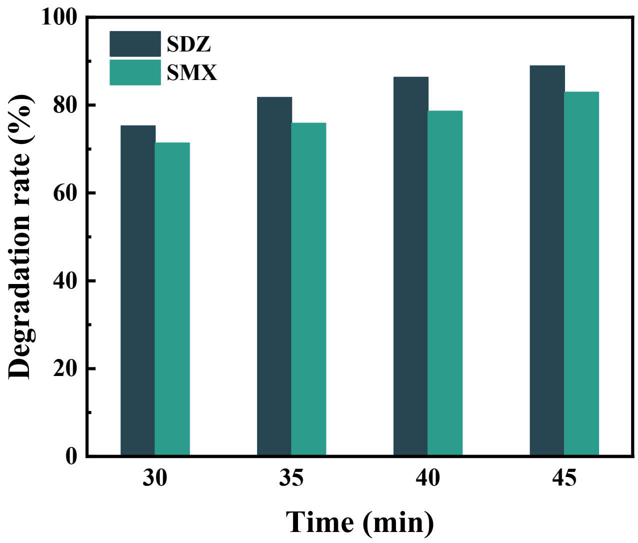

Figure S8. Degradation rates of SDZ and SMX after 30 min, conditions: $[TC]_0$= 10 μM, $[PAA]_0$= 100 μM, $[BNQDs\text{-}FCN]_0$ = 0.2 g/L, $[pH]_0$ = 7 ± 0.2.

Atom	Fukui (-)	Fukui (+)	Fukui (0)
C (1)	0.008	0.009	0.009
C (2)	0.028	0.01	0.019
C (3)	0.013	0.009	0.011
C (4)	0.025	0	0.012
C (5)	0.002	0.003	0.003
C (6)	0.034	0.007	0.02
C (7)	-0.001	-0.007	-0.004
C (8)	-0.003	-0.011	-0.007
C (9)	0.006	0.017	0.012
C (10)	0.012	0.015	0.014
C (11)	0.02	-0.003	0.009
C (12)	-0.005	-0.01	-0.007
C (13)	-0.009	-0.01	-0.01
C (14)	-0.011	-0.01	-0.01
C (15)	-0.013	-0.017	-0.015
C (16)	0	0.11	0.055
C (17)	0.012	0.001	0.007
C (18)	0.004	0.094	0.049
O (19)	0.075	0.027	0.051
O (20)	0.024	0.024	0.024
O (21)	0.029	0.021	0.025
O (22)	0.016	0.031	0.023
O (23)	0.025	0.1	0.063
O (24)	-0.007	0.078	0.036
C (25)	0.009	0.02	0.015
N (26)	-0.001	-0.004	-0.003
O (27)	0.031	0.049	0.04
N (28)	0.095	-0.01	0.043
C (29)	-0.029	-0.017	-0.023
C (30)	-0.028	-0.017	-0.022
O (31)	0.011	0.014	0.012
C (32)	-0.011	-0.008	-0.009
H (33)	0.011	0.021	0.016
H (34)	0.045	0.031	0.038
H (35)	0.047	0.03	0.038
H (36)	0.041	0.022	0.032
H (37)	-0.01	0.01	0
H (38)	0.027	0.026	0.027
H (39)	0.007	0.025	0.016
H (40)	0.025	0.021	0.023
H (41)	0.025	0.035	0.03
H (42)	0.016	0.01	0.013
H (43)	0.014	0.014	0.014
H (44)	0.015	0.02	0.018
H (45)	0.006	0.018	0.012
H (46)	0.021	0.038	0.03
H (47)	0.058	0.023	0.04
H (48)	0.052	0.03	0.041
H (49)	0.04	0.009	0.025
H (50)	0.042	0	0.021
H (51)	0.045	0.032	0.039
H (52)	0.055	0.023	0.039
H (53)	0.013	0.005	0.009
H (54)	0.022	0.023	0.022
H (55)	0.001	0.008	0.004
H (56)	0.02	0.009	0.015

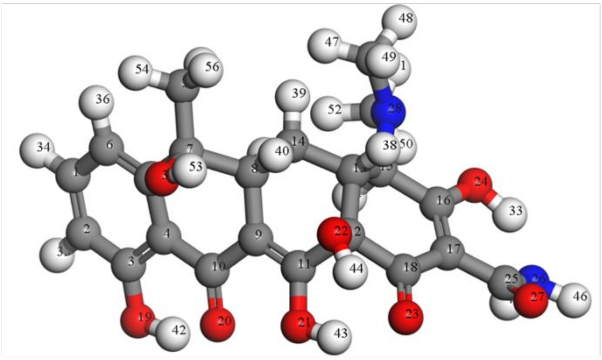

Figure S9. The Fukui index of atoms in TC molecule.

Atom	Fukui (-)	Fukui (+)	Fukui (0)
C (1)	0.012	0.009	0.01
C (2)	0.049	0.009	0.029
C (3)	0.021	0.008	0.015
C (4)	0.05	0	0.025
C (5)	0.01	0.002	0.006
C (6)	0.066	0.006	0.036
C (7)	-0.002	-0.006	-0.004
C (8)	-0.006	-0.01	-0.008
C (9)	0.013	0.017	0.015
C (10)	0.019	0.014	0.017
C (11)	0.035	-0.001	0.017
C (12)	-0.005	-0.005	-0.005
C (13)	-0.008	-0.01	-0.009
C (14)	-0.005	-0.004	-0.004
C (15)	0.002	-0.023	-0.01
C (16)	0.005	0.114	0.059
C (17)	0.005	0.006	0.006
C (18)	0.001	0.087	0.044
O (19)	0.127	0.026	0.076
O (20)	0.031	0.023	0.027
O (21)	0.04	0.02	0.03
O (22)	0.024	0.017	0.02
O (23)	0.017	0.1	0.059
O (24)	0.009	0.072	0.041
C (25)	0.006	0.026	0.016
N (26)	0	-0.004	-0.002
O (27)	0.02	0.057	0.038
N (28)	-0.002	0.003	0.001
C (29)	-0.011	-0.023	-0.017
C (30)	-0.004	-0.019	-0.011
O (31)	0.008	0.015	0.011
C (32)	-0.014	-0.007	-0.01
H (33)	0.008	0.021	0.015
H (34)	0.066	0.03	0.048
H (35)	0.069	0.029	0.049
H (36)	0.07	0.021	0.046
H (37)	0.014	0.009	0.012
H (38)	0.027	0.023	0.025
H (39)	0.018	0.024	0.021
H (40)	-0.012	0.04	0.014
H (41)	0.023	0.01	0.016
H (42)	0.016	0.015	0.016
H (43)	0.007	0.021	0.014
H (44)	0.003	0.02	0.011
H (45)	0.016	0.04	0.028
H (46)	0.015	0.036	0.026
H (47)	0.022	0.01	0.016
H (48)	0.007	0.014	0.01
H (49)	0.022	0.013	0.018
H (50)	0.015	0.033	0.024
H (51)	-0.012	0.012	0
H (52)	0.018	0.006	0.012
H (53)	0.015	0.023	0.019
H (54)	0.016	0.006	0.011
H (55)	0.034	0.008	0.021
O (56)	-0.006	0.001	-0.002
H (57)	0.017	0.015	0.016

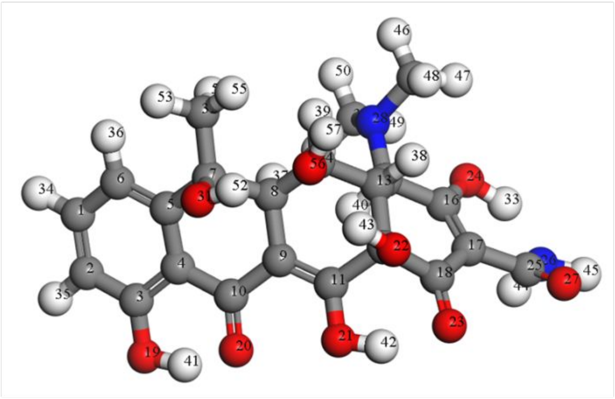

Figure S10. The Fukui index of atoms in OTC molecule.

Atom	Fukui (-)	Fukui (+)	Fukui (0)
O (1)	0.039	0.02	0.03
C (2)	0.013	0.008	0.011
C (3)	0.016	0.004	0.01
C (4)	0.017	-0.002	0.007
N (5)	0.055	0.008	0.032
C (6)	-0.004	-0.003	-0.004
N (7)	0.047	-0.008	0.02
S (8)	0.014	0.057	0.035
C (9)	0.025	0.037	0.031
O (10)	0.06	0.057	0.059
O (11)	0.068	0.061	0.065
C (12)	0.013	0.085	0.049
C (13)	0.031	0.008	0.019
C (14)	0.012	0.076	0.044
C (15)	0.039	0.038	0.039
C (16)	0.014	0.029	0.022
N (17)	0.091	0.049	0.07
H (18)	0.036	0.018	0.027
H (19)	0.023	0.013	0.018
H (20)	0.027	0.017	0.022
H (21)	0.028	0.019	0.023
H (22)	0.031	0.014	0.023
H (23)	0.043	0.075	0.059
H (24)	0.058	0.077	0.068
H (25)	0.062	0.077	0.069
H (26)	0.047	0.067	0.057
H (27)	0.047	0.048	0.047
H (28)	0.046	0.05	0.048

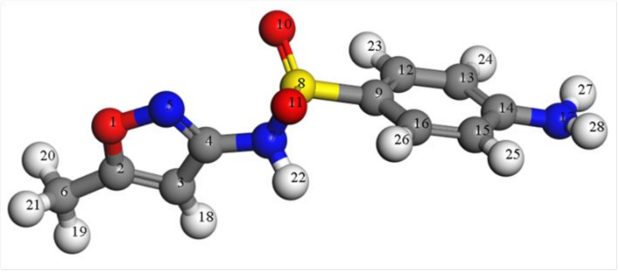

Figure S11. The Fukui index of atoms in SMX molecule.

Atom	Fukui (-)	Fukui (+)	Fukui (0)
C (1)	0.038	0.056	0.047
C (2)	0.036	0.125	0.08
N (3)	0.067	0.109	0.088
C (4)	0.019	0.04	0.029
N (5)	0.072	0.093	0.083
C (6)	0.034	0.132	0.083
N (7)	0.01	0.022	0.016
S (8)	0.016	0.01	0.013
O (9)	0.04	0.025	0.033
O (10)	0.041	0.025	0.033
C (11)	0.05	0	0.025
C (12)	0.029	0.014	0.022
C (13)	0.049	0.017	0.033
C (14)	0.037	0.022	0.03
C (15)	0.051	0.019	0.035
C (16)	0.029	0.006	0.018
N (17)	0.103	0.024	0.063
H (18)	0.028	0.043	0.035
H (19)	0.031	0.062	0.046
H (20)	0.033	0.063	0.048
H (21)	0.011	0.021	0.016
H (22)	0.021	0.008	0.015
H (23)	0.028	0.014	0.021
H (24)	0.029	0.015	0.022
H (25)	0.021	0.006	0.013
H (26)	0.039	0.014	0.027
H (27)	0.039	0.014	0.027

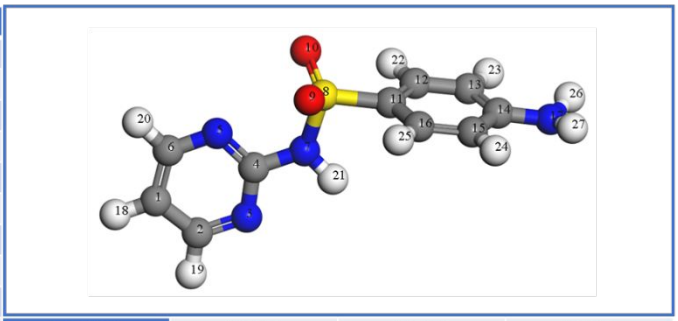

Figure S12. The Fukui index of atoms in SDZ molecule.

Atom	Fukui (-)	Fukui (+)	Fukui (0)
N (1)	0.057	0.019	0.038
C (2)	0.018	0.006	0.012
C (3)	0.018	0.006	0.012
N (4)	0.064	0.013	0.039
C (5)	0.019	0.007	0.013
C (6)	0.018	0.005	0.012
C (7)	0.018	0.031	0.025
C (8)	0.03	0.042	0.036
C (9)	0.02	0.012	0.016
C (10)	0.038	0.008	0.023
C (11)	0.022	0.045	0.034
C (12)	0.031	0.047	0.039
C (13)	0.031	0.038	0.035
C (14)	0.018	0.047	0.033
C (15)	0.024	0.11	0.067
N (16)	0.012	0.034	0.023
C (17)	-0.001	0.001	0
C (18)	0.005	0.014	0.009
C (19)	0.013	0.014	0.013
O (20)	0.123	0.069	0.096
C (21)	0.014	0.034	0.024
O (22)	0.024	0.025	0.025
O (23)	0.039	0.065	0.052
F (24)	0.028	0.036	0.032
H (25)	0.028	0.018	0.023
H (26)	0.016	0.003	0.01
H (27)	0.016	0.003	0.01
H (28)	0.026	0.013	0.019
H (29)	0.027	0.011	0.019
H (30)	0.018	0.004	0.011
H (31)	0.027	0.015	0.021
H (32)	0.016	0.005	0.01
H (33)	0.029	0.015	0.022
H (34)	0.014	0.019	0.017
H (35)	0.02	0.026	0.023
H (36)	0.018	0.041	0.03
H (37)	0.009	0.02	0.014
H (38)	0.008	0.018	0.013
H (39)	0.002	0.008	0.005
H (40)	0.014	0.019	0.017
H (41)	0.008	0.01	0.009
H (42)	0.021	0.027	0.024
H (41)	0.008	0.01	0.024
H (42)	0.021	0.027	0.038

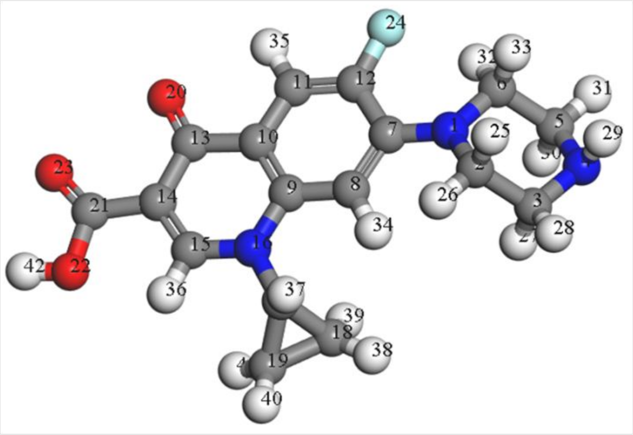

Figure S13. The Fukui index of atoms in CIP molecule.

Atom	Fukui (-)	Fukui (+)	Fukui (0)
N (1)	0.1	0.014	0.057
C (2)	-0.027	-0.007	-0.017
C (3)	-0.028	-0.02	-0.024
N (4)	0.066	0.021	0.043
C (5)	-0.031	-0.019	-0.025
C (6)	-0.026	-0.01	-0.018
C (7)	-0.005	0.032	0.013
C (8)	0.027	0.036	0.031
C (9)	0.021	0.063	0.042
C (10)	0.032	-0.007	0.013
C (11)	0.018	0.012	0.015
C (12)	0.029	0.048	0.038
N (13)	0	0.005	0.002
C (14)	0.012	0.09	0.051
C (15)	0.011	0.018	0.014
C (16)	0.018	0.053	0.035
F (17)	0.024	0.036	0.03
C (18)	-0.014	-0.031	-0.022
C (19)	-0.008	-0.018	-0.013
O (20)	0.069	0.088	0.078
C (21)	0.012	0.019	0.015
O (22)	0.021	0.031	0.026
O (23)	0.029	0.049	0.039
H (24)	0.051	0.02	0.036
H (25)	0.043	0	0.021
H (26)	0.066	0.036	0.051
H (27)	0.059	0.036	0.048
H (28)	0.05	0.012	0.031
H (29)	0.058	0.039	0.048
H (30)	0.056	0.01	0.033
H (31)	0.065	0.034	0.049
H (32)	0.04	0	0.02
H (33)	0.04	0.05	0.045
H (34)	0.02	0.04	0.03
H (35)	0.032	0.056	0.044
H (36)	0.005	0.037	0.021
H (37)	0.01	0.035	0.022
H (38)	0.015	0.017	0.016
H (39)	0.014	0.018	0.016
H (40)	0.02	0.038	0.029
H (41)	0.008	0.02	0.014

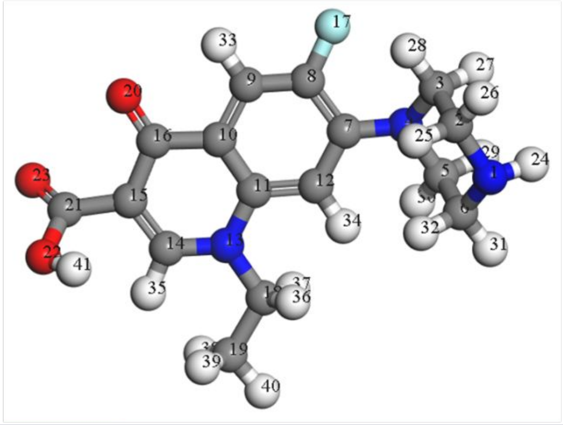

Figure S14. The Fukui index of atoms in NOR molecule.

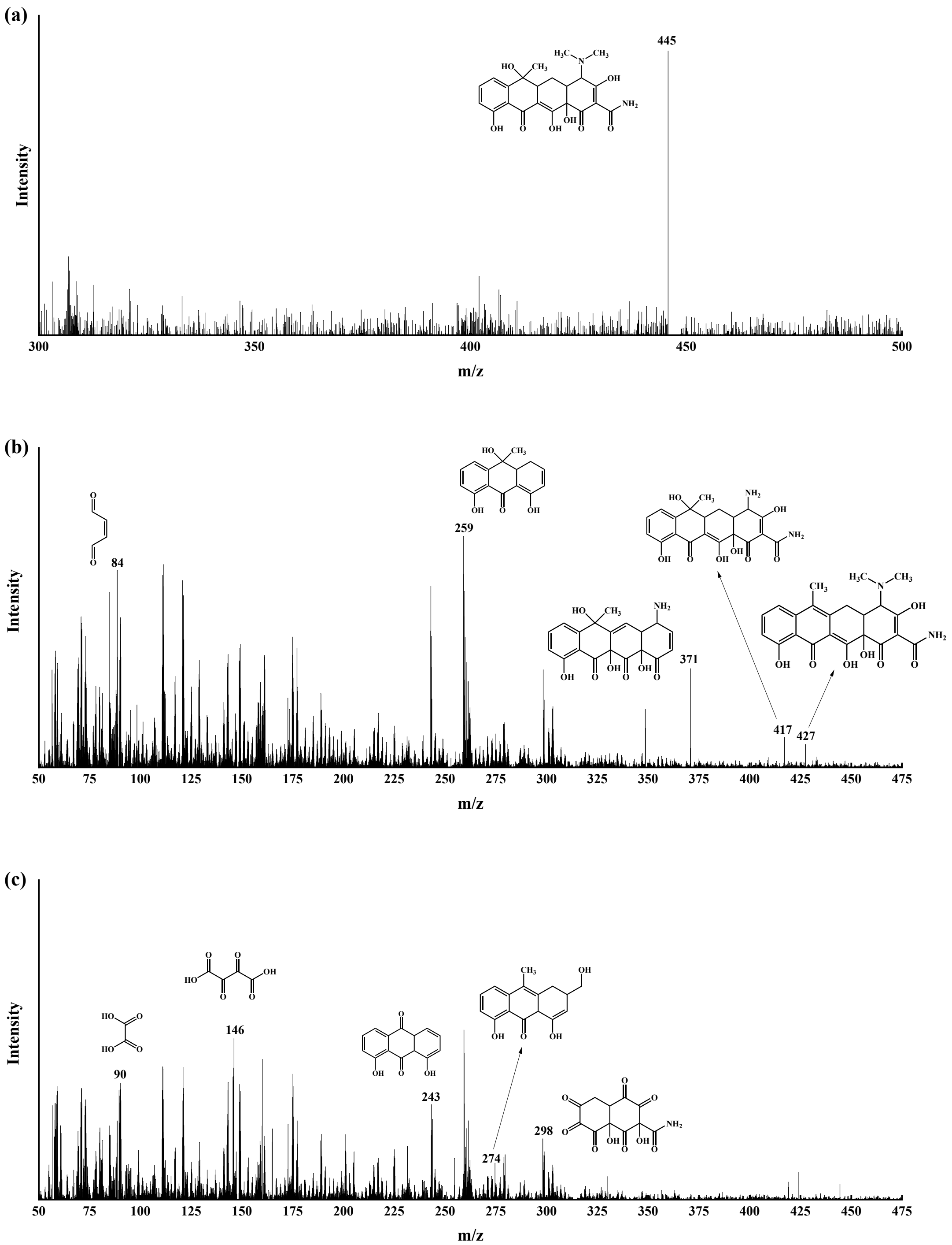

Figure S15. Mass spectra of intermediates at different reaction times: (a) 0 min, (b) 15 min, and (c) 30 min.

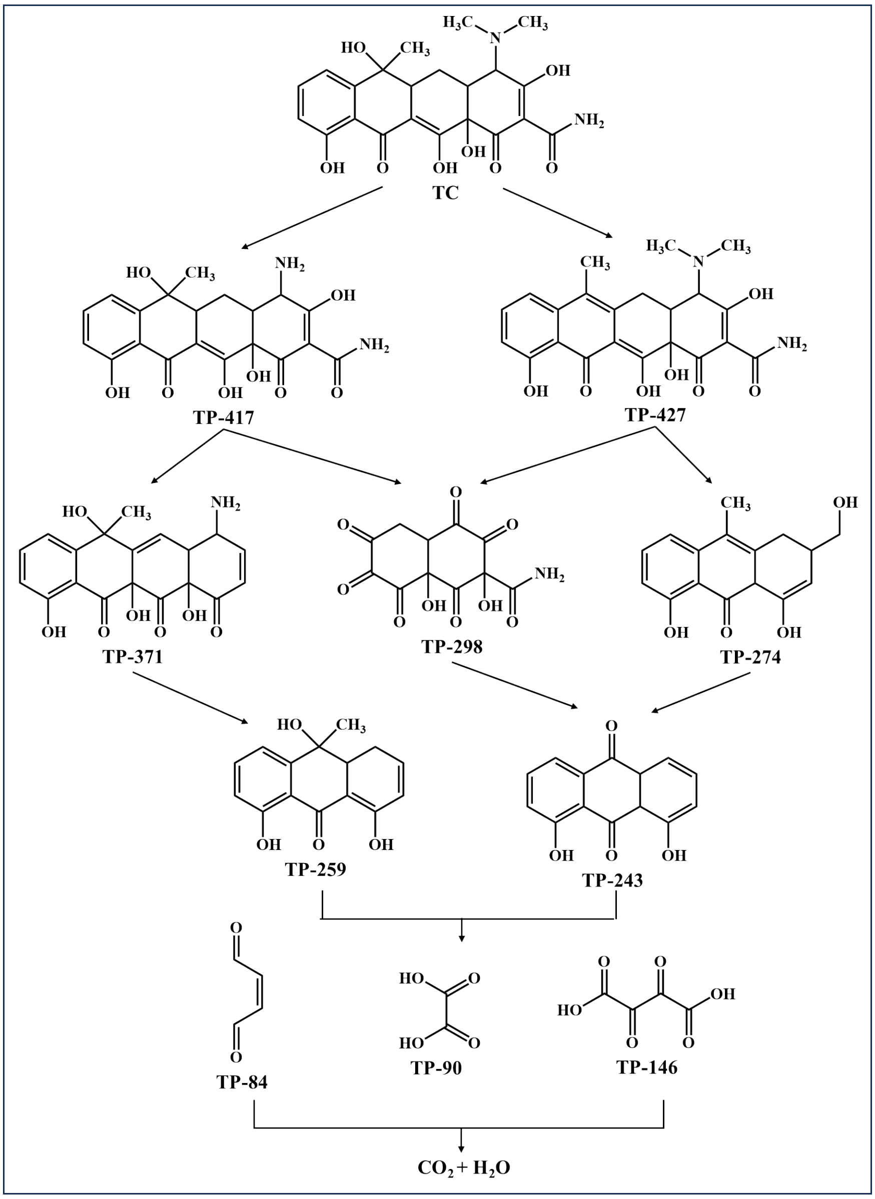

Figure S16. Degradation pathways of TC.

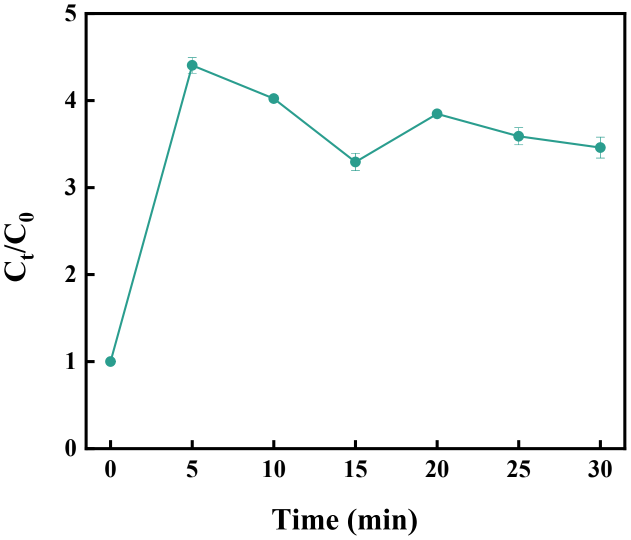

Figure S17. Change of TOC in the BNQDs-FCN/PAA system, conditions: $[TC]_0 = 10$ μM, $[PAA]_0 = 100$ μM, $[BNQDs\text{-}FCN]_0 = 0.2$ g/L, $[pH]_0 = 7 \pm 0.2$.

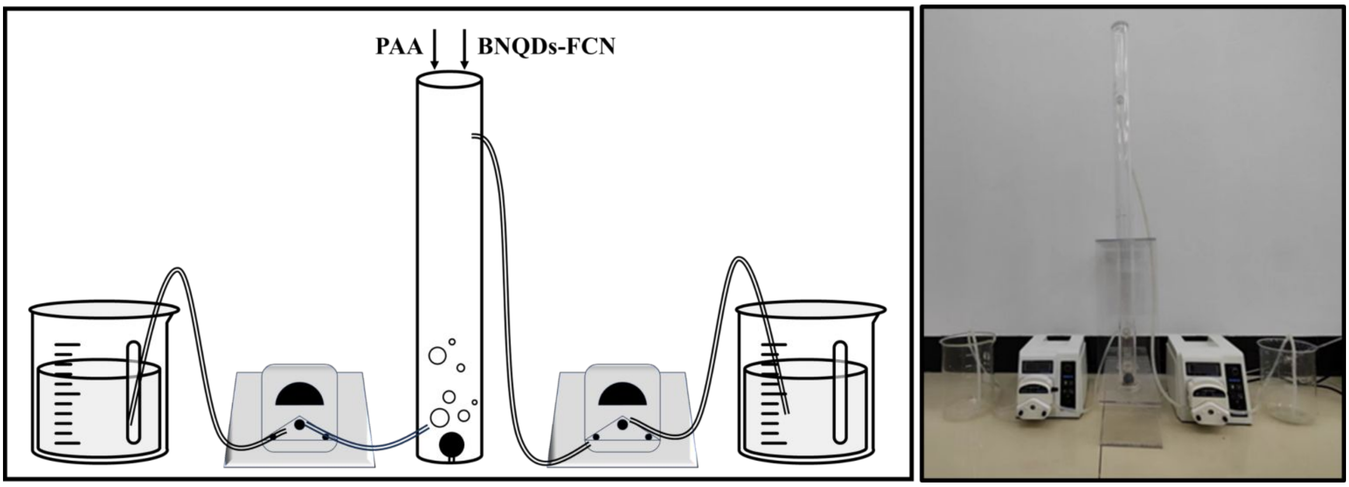

Figure S18. Process flow chart.

Table S1. EXAFS fitting parameters at the Fe K-edge for BNQDs-FCN.

Sample	Shell	N	R (Å)	σ^2 (Å^2)	R factor (%)
BNQDs-FCN	Fe-N	3.99	1.93	0.0101	0.016

Table S2. Prediction of acute and chronic toxicity of TC and intermediates in the BNQDs-FCN/PAA systems by ECOSAR.

	Fish		Daphnid		Green Algae	
Products	96 h-LC_{50} (mg/L)	Chv (mg/L)	48 h-LC_{50} (mg/L)	Chv (mg/L)	96 h-EC_{50} (mg/L)	Chv (mg/L)
TC	226	1.29	5.3	1.25	23.8	3.28
TP-417	1.97E+04	4.15E+03	1.54E+03	83.7	2.93E+03	717
TP-427	750	73.3	75.4	5.25	87.1	25.6
TP-371	203	15.1	22.4	1.7	21.6	6.77
TP-298	1.15E+09	5.00E+07	3.29E+08	4.75E+06	1.44E+07	8.16E+05
TP-274	29.5	3.21	18.3	2.3	19.8	6.36
TP-259	48.7	5.14	29.5	3.46	28.8	8.74
TP-243	468	43.8	256	22.4	163	39.2
TP-122	32.1	3.33	19.2	2.15	17.6	5.17
TP-90	1.68E+05	1.09E+04	6.75E+04	2.52E+03	1.21E+04	1.47E+03
TP-84	3.27E+03	261	1.56E+03	94.3	571	102
TP-146	1.50E+04	1.14E+03	6.89E+03	368	2.10E+03	341

Table S3. Comparison of pollutant degradation levels by different Fe-N catalysts.

Catalyst	Reaction time (min)	Pollutant	Initial concentration	Degradation rate constant (min^{-1})	Reference
Fe_5-NC	60	Oxalic acid (OA)	100 mg/L	0.040	[1]
5%Fe-g-C_3N_4	30	Metronidazole (MNZ)	10 mg/L	0.0643	[2]
Fe-N_4-C_x (1200:1)	120	Enrofloxacin (ENR)	10 mg/L	0.0278	[3]
FeB_5-g-C_3N_4	60	TC	10 mg/L	0.0368	[4]
BNQDs-FCN	30	TC	10 μM	0.0843	This study

References

(1) Ren, T.; Chen, S.; Yin, M.; Ouyang, C.; Huang, X.; Zhang, X. Single-Atom Fe-N4 Sites for Catalytic Ozonation to Selectively Induce a Nonradical Pathway toward Wastewater Purification. *Environmental Science And Technology* **2023**, *57* (9), 3623-3633.

(2) Li, Q.; Xu, H.; Zhou, G.; Cheng, F.; Wang, Q. Sulfite activation by Fe-doped g-C3N4 for metronidazole degradation. *Separation and Purification Technology* **2021**, *272*, 118928.

(3) Wang, Y.; Xiao, T.; Zuo, S.; Wan, J.; Yan, Z.; Bin, Z.; Zhang, X. Exploring degradation properties and mechanisms of emerging contaminants via enhanced directional electron transfer by polarized electric fields regulation in Fe-N4-Cx. *Journal of Hazardous Materials* **2023**, *446*, 130698.

(4) Xu, Z.; Wu, M.; Gao, Z.; Wang, Y.; Guo, Y.; Shen, C.; Zeng, X.; Li, F.; You, Z.; He, S.; et al. Insights into the coordination-driven role of boron in Fe-doped carbon nitride composites activation of peracetic acid to remove tetracycline: Function and contribution of boron. *Journal of Environmental Chemical Engineering* **2024**, *12* (1), 111674.
